# The arginine deaminase system plays distinct roles in *Borrelia burgdorferi* and *Borrelia hermsii*

**DOI:** 10.1371/journal.ppat.1010370

**Published:** 2022-03-14

**Authors:** Crystal L. Richards, Sandra J. Raffel, Sébastien Bontemps-Gallo, Daniel P. Dulebohn, Tessa C. Herbert, Frank C. Gherardini

**Affiliations:** 1 Laboratory of Bacteriology, Gene Regulation Section, Division of Intramural Research, Rocky Mountain Laboratories, National Institute of Allergy and Infectious Diseases, National Institutes of Health, Hamilton, Montana, United States of America; 2 Univ. Lille, CNRS, Inserm, CHU Lille, Institut Pasteur de Lille, U1019-UMR9017-CIIL-Centre d’Infection et d’Immunité de Lille, Lille, France; 3 University of Colorado Hospital, Molecular Diagnostics Laboratory, Aurora, Colorado, United States of America; Medical College of Wisconsin, UNITED STATES

## Abstract

*Borrelia* species are amino acid auxotrophs that utilize di- and tri- peptides obtained through their oligopeptide transport system to supply amino acids for replicative growth during their enzootic cycles. However, *Borrelia* species from both the Lyme disease (LD) and relapsing fever (RF) groups harbor an amino acid transport and catabolism system, the Arginine Deiminase System (ADI), that could potentially augment intracellular L-arginine required for growth. RF spirochetes contain a “complete”, four gene ADI (*arcA*, *B*, *D*, and *C*) while LD spirochetes harbor *arcA*, *B*, and sometimes *D* but lack *arcC* (encoding carbamate kinase). In this study, we evaluated the role of the ADI system in bacterial survival and virulence and discovered important differences in RF and LD ADIs. Both in vitro and in a murine model of infection, *B*. *hermsii* cells significantly reduced extracellular L-arginine levels and that reduction was dependent on arginine deiminase expression. Conversely, *B*. *burgdorferi* did not reduce the concentration of L-arginine during in vitro growth experiments nor during infection of the mammalian host, suggesting a fundamental difference in the ability to directly utilize L-arginine compared to *B*. *hermsii*. Further experiments using a panel of mutants generated in both *B*. *burgdorferi* and *B*. *hermsii*, identified important differences in growth characteristics and ADI transcription and protein expression. We also found that the ADI system plays a key role in blood and spleen colonization in RF spirochetes. In this study we have identified divergent metabolic strategies in two closely related human pathogens, that ultimately impacts the host-pathogen interface during infection.

## Author summary

Reports of tick-borne diseases have been steadily increasing in the US and the number of Lyme disease cases caused by *B*. *burgdorferi* have tripled since the late 1990’s. Although less common, cases of tick-borne relapsing fever, caused by *B*. *hermsii* and *B*. *turicatae* in the US, have increased as well. While transmitted by different ticks and maintained in unique enzootic cycles, the closely related spirochetes *B*. *burgdorferi* and *B*. *hermsii* share numerous genetic features including a truncated and streamlined capacity for metabolic activity. In this study we combine genetic and biochemical assays to define the role of the ADI in the infective cycles of *B*. *burgdorferi* and *B*. *hermsii*. When we compared *B*. *burgdorferi* and *B*. *hermsii*, we identified important differences in their respective ADI’s including operon arrangement, sensitivity to L-arginine and L-ornithine levels, as well as gene and protein expression. In addition, we show that arginine deiminase is required to reduce host L-arginine levels during murine infection with *B*. *hermsii*. This study provides new insights into the metabolic activities of two medically relevant spirochetes and highlights the dynamic nature of host-pathogen interactions.

## Introduction

Bacterial pathogens have evolved to take advantage of the nutrient rich environments encountered in their various hosts. In some cases, pathogens produce effectors that impact the host and facilitate infection [1–6]. In other cases, metabolic activities can alter the bacteria-host interface to give the pathogen an advantage [7–11]. One such bacterial system is the arginine deiminase system (ADI), a secondary catabolic pathway that facilitates the import of L-arginine and export of L-citrulline and/or L-ornithine, followed by the catabolic breakdown of L-arginine and generation of ATP, CO_2_ and NH_3_^+^. ADIs have been described in several species of bacteria and have been shown to play an important role in growth, virulence, and resistance to environmental stresses [12–18]. During some bacterial infections, it has been shown that the ability of a pathogen to utilize L-arginine can influence disease progression and severity [10,19–23]. For example, *Streptococcus pyogenes* mutants deficient in either arginine deiminase or ornithine transcarbamylase are attenuated or avirulent, respectively, in a murine mucosal infection model [10,13]. The pathogen, *Staphylococcus aureus* harbors a constitutively active *arc* operon (denoted ACME) that has been shown to directly affect the host’s production of polyamines by modulating the inducible nitric oxide synthase (iNOS)/arginase pathways in innate immune cells [24].

The utilization and sequestration of metabolites is critically important for vector-borne pathogens as they experience dramatic changes in nutrient availability and environmental stresses when they shuttle between vertebrate mammals and blood-feeding arthropods [8,25–30]. Relapsing fever (RF) and Lyme disease (LD) spirochetes are pathogens reliant upon blood feeding arthropods and mammalian hosts to be maintained in their respective natural life cycles. The obligate parasites, *Borrelia hermsii* (Bh) and *Borrelia burgdorferi* (Bb), have a reduced metabolic biosynthetic capacity compared to most bacterial species and have adapted to obtain most of their cellular building blocks directly from their respective host(s). The ADI appears to be conserved in all relapsing fever spirochetes (RFSs) and is the only secondary catabolic pathway capable of producing ATP. All RFS genomes sequenced to date contain genes for the complete *arc* operon, encoding the entire core set of cytoplasmic L-arginine degrading enzymes *arcA*, *B and C*. However, all Lyme disease spirochetes (LDSs) appear to be missing various components; carbamate kinase (*arcC*) has not been identified in any LDS genome. This difference is noteworthy as ArcC is the ATP-generating enzyme of the ADI and its expression allows RFS to generate ATP from L-arginine.

Relapsing fever is an acute febrile disease characterized by recurrent bacteremia and is caused by primarily two species of *Borrelia* in North America, *Borrelia hermsii* and *Borrelia turicatae* [31,32]. These spirochetes survive in and are transmitted by *Ornithodoros* tick species, *Ornithodoros hermsi* and *Ornithodoros turicata*, respectively [33–36]. Upon transmission of a RFS by tick bite, the disease is caused by rapid multiplication of bacteria in the host mammal’s blood, with numbers reaching as high as 10^8^ spirochetes per ml with an accompanying inflammatory response in the host [37]. Relapsing fever is quite distinct from Lyme disease, which is an invasive infection with spirochetes disseminating throughout mammalian tissues such as the cartilaginous joints, skin, spleen, liver, heart, adipose, uterus and lymph nodes [36,38,39]. LDS are rarely observed in the blood during an initial infection and localize to immune privileged sites to take up long term residence [40]. In the United States, the primary agent of Lyme disease is *Borrelia* (*Borreliella*) *burgdorferi* and it is the most common tick-borne disease in North America [41–44].

In the present work, we examined the role of the ADI in the infective cycles of the RFS and LDS, *B*. *hermsii* and *B*. *burgdorferi*, respectively. In a series of experiments, the relationship between extracellular L-arginine, spirochetal physiology, and virulence was examined. Analysis of *arc* mutants in both *B*. *burgdorferi* and *B*. *hermsii* led to several discoveries. First, the ADI system was not required for growth or spirochetal replication in either spirochetal species in vitro. Second, the expression patterns of *B*. *burgdorferi* ADI genes and proteins were considerably different than those observed in *B*. *hermsii*, highlighting a significant difference in the regulation of the ADI. Third, arginine deiminase expression was required for L-arginine utilization both in vitro and in vivo. Our results also showed that the utilization of L-arginine promoted the survival of WT *B*. *hermsii* in vivo. Significantly lower levels of viable bacteria were cultured from the blood and spleens of mice infected with the *B*. *hermsii* arginine deiminase mutant. These experiments highlight complex inter-relatedness of host-pathogen interactions and shed light on key metabolic differences in LDS and RFS.

## Results

### The ADI is a highly conserved amino acid catabolic system in both Lyme disease and relapsing fever spirochetes

**Fig 1:**
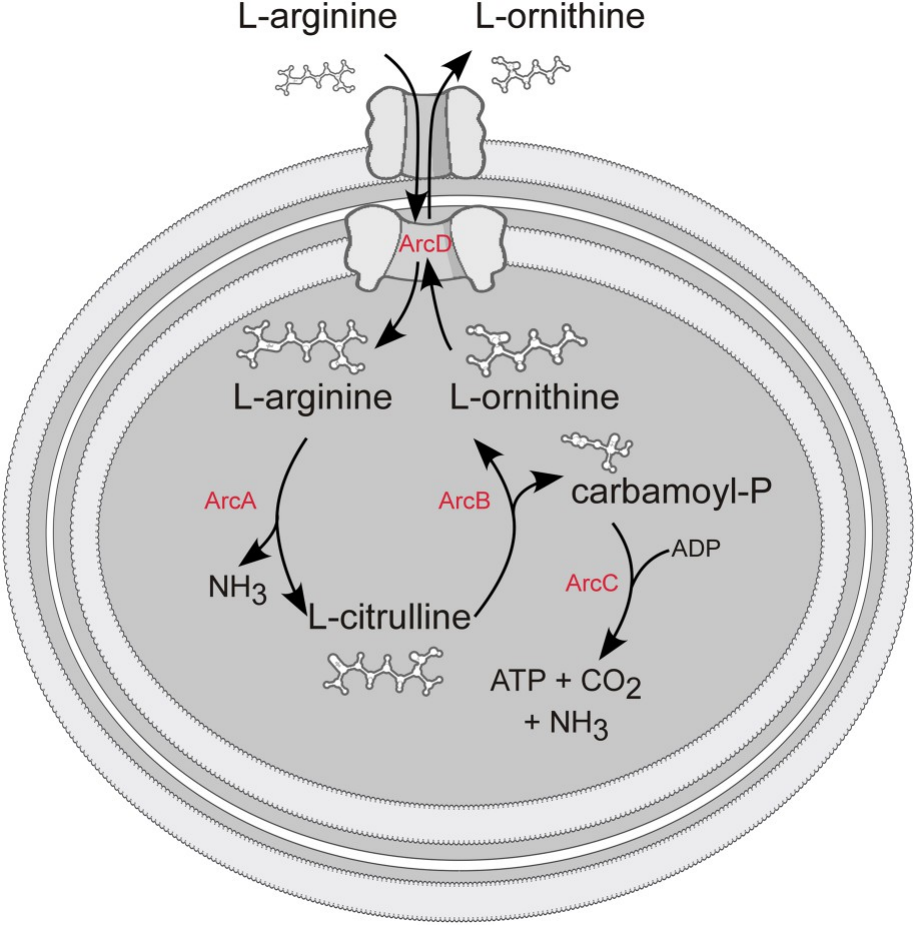
The bacterial arginine deiminase system (ADI) facilitates the transport and catabolism of L-arginine. Bacterial ADIs use the antiporter, ArcD, to import L-arginine and export L-ornithine. Once inside the cell, L-arginine is broken down into L-citrulline by arginine deiminase (ArcA), then to L-ornithine by ornithine transcarbamylase (ArcB). Carbamate kinase (ArcC) converts carbamoyl phosphate to ammonia, carbon dioxide and ATP.

**Fig 2:**
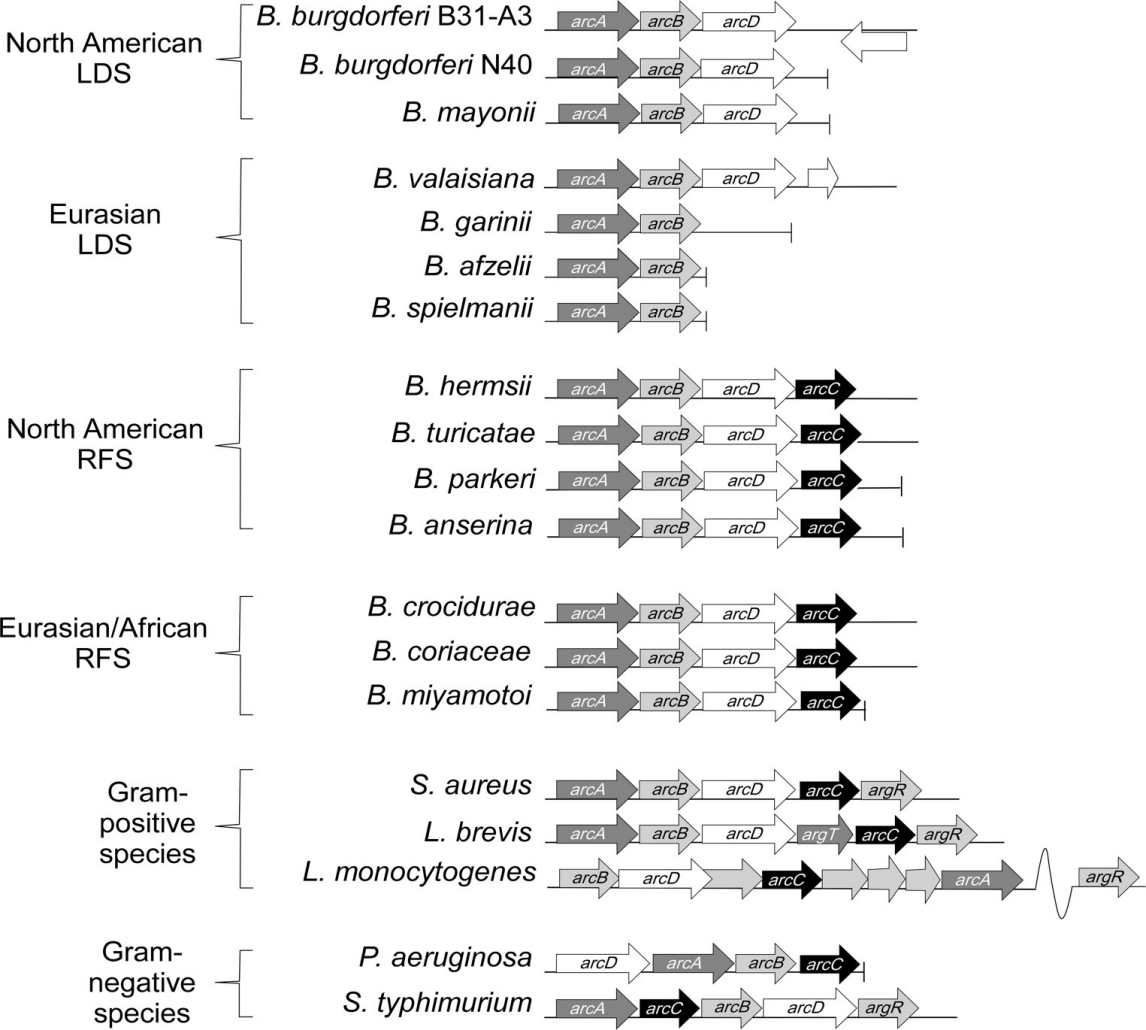
The ADI operon composition and organization varies among bacteria. Lyme disease spirochete genomes have ADI operons that contain only *arcAB* or *arcABD* while all relapsing fever spirochete genomes sequenced to date contain *arcABDC* [28, 53].

*Borrelia* species have evolved streamlined metabolic capabilities that obligate the bacteria to acquire most of their cellular building blocks directly from their tick and mammalian hosts [45]. Within this group of spirochetes, the ADI is the only secondary catabolic pathway that has been identified with the potential to contribute to both growth and energy production. Bacterial ADIs typically contain a dedicated antiporter, that imports L-arginine and exports L-citrulline and/or L-ornithine. ADIs also typically contain three core enzymes that sequentially break down L-arginine into the end products L-ornithine, ATP, carbon dioxide, and ammonia (Fig 1) [46–52]. Fig 2 shows the ADI operon composition and gene organization for several species of LDSs, RFSs, and other bacterial species that contain an experimentally characterized ADI (*Pseudomonas aeruginosa*, *Salmonella enterica* subsp. *Typhimurium*, and *Listeria monocytogenes*). Genes encoding arginine deiminase, ArcA, (*bb0841*/*bh0841*) and ornithine transcarbamylase, ArcB (*bb0842*/*bh0842*) have been identified in the genomes of both *B*. *burgdorferi* and *B*. *hermsii*, respectively (Fig 2) [28]. However, no gene encoding a homolog to the third catabolic enzyme, carbamate kinase, ArcC has been identified in any LDS sequenced to date but has been identified in all RFS, including *B*. *hermsii*, (*arcC*, *bh0843A*) (Fig 2) [45,51]. All the North American LDS and RFS species analyzed in this study contained homologs to the antiporter ArcD (*arcD*, *bb0843*/bh0843). However, *arcD* was not identified in the genomes of any of the European/Eurasian Lyme disease strains analyzed (Fig 2) [53].

Phylogenetic analysis (RAxML; bootstrap analysis 1000 replicates) of up to 19 LDS and RFS strains (plus several representative Gram-negative and Gram-positive bacterial strains) revealed the genetic relatedness of each ADI gene (S1–S4 Figs) [54]. A comparison of the arginine deiminase amino acid sequences from *B*. *burgdorferi* and *B*. *hermsii* revealed significant variation in the sequence identity (70.3% identity conservation). Similarly, ornithine transcarbamylase amino acid sequences were 74.6% identical in *B*. *burgdorferi* compared to *B*. *hermsii*. In each analysis, the backbone topologies were poorly resolved (<50%); however, clades with strong support, including branches showing LDS and RFS clades distinct from the other bacterial sequences, were identified (S1–S3 Figs). Analysis of 15 carbamate kinase (*arcC*) sequences (10 RFS, 5 other) showed a general conservation of sequence identity among RFSs (S4 Fig). Compared to *B*. *hermsii* strain DAH (used in this study), *B*. *hermsii* strains MTW and HS1 have 95.2% and 100% identity conservation, respectively. Grouping of RFS carbamate kinase sequences is consistent with their geographic location, with North American RFS forming one group and European/African RFS forming another.

Analysis of the putative L-arginine/L-ornithine antiporter revealed *arcD* has a lower level of amino acid sequence identity (67.9% identity) when comparing *B*. *burgdorferi* and *B*. *hermsii*, than the other conserved genes in the operon. In contrast to the tree topologies observed with ArcA and ArcB, analysis of ArcD sequences showed a highly resolved clade among the North American LDSs and the non-spirochetal bacterial sequences included in the study (S3 Fig). Previous studies have attempted to delineate the evolutionary history of the ADI in both eukaryotes and prokaryotes and have shown that each gene in the ADI gene cluster is under selective pressure and can evolve at different rates [50–52]. The results of this study further support those conclusions and highlight the evolutionary divergence of each individual ADI gene as it corresponds to LDS and RFS phylogenetic groupings.

### *B*. *burgdorferi* and *B*. *hermsii* have different growth and ADI expression patterns during in vitro cultivation in the presence of L-arginine and L-ornithine

**Fig 3:**
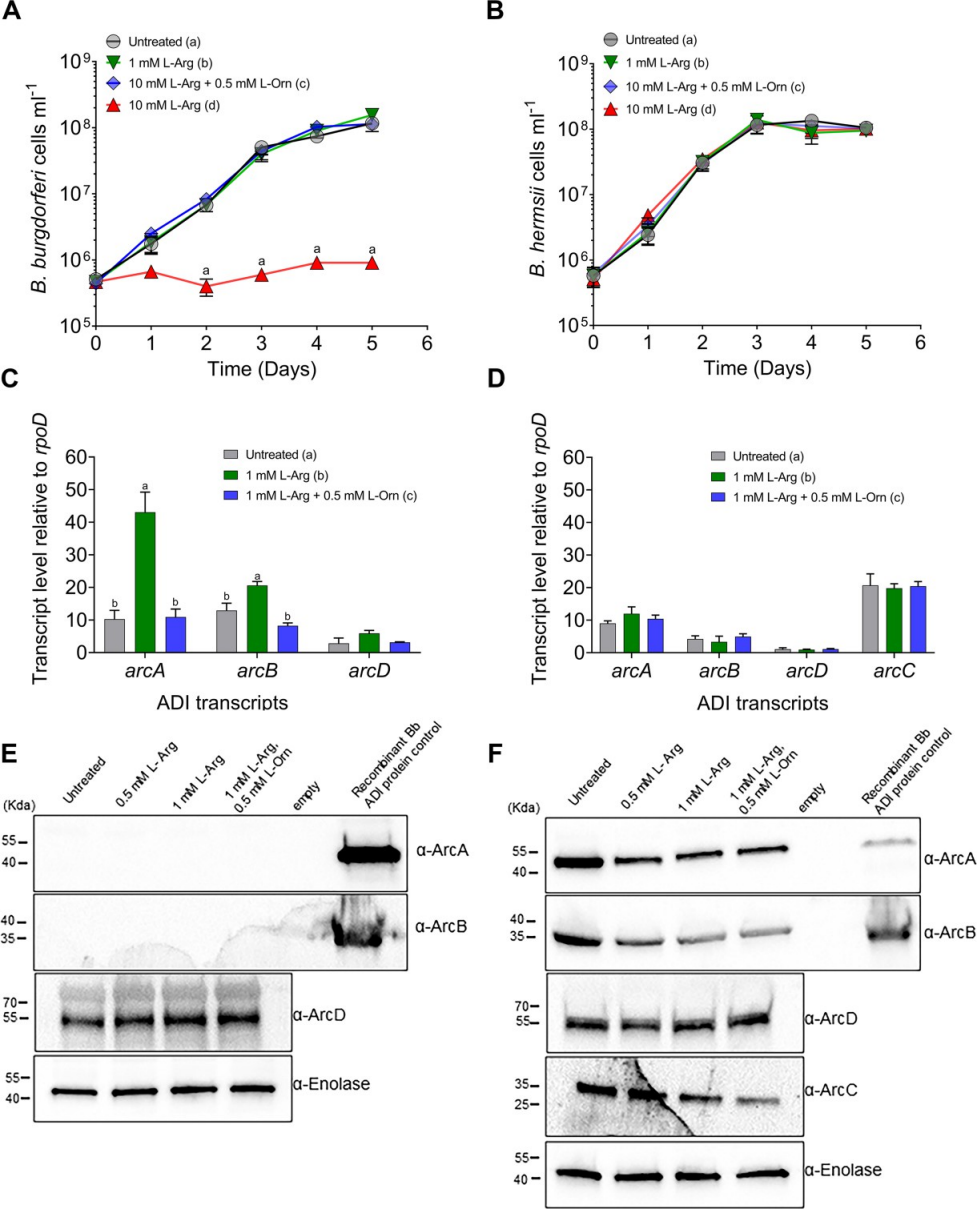
*B*. *burgdorferi* and *B*. *hermsii* respond differently to the presence of L-arginine and L-ornithine during in vitro growth. *B*. *burgdorferi* (A) and *B*. *hermsii* (B) were grown in the presence of exogenous L-arginine or L-arginine plus L-ornithine. Growth was monitored by dark field microscopy. Statistical analysis was performed using a repeated measures ANOVA with Dunnett correction for multiple comparison. Letters indicate p-value < 0.05 in multiple comparison testing. *B*. *burgdorferi* (C) and *B*. *hermsii* (D) were grown in the presence of exogenous L-arginine or L-arginine and L-ornithine combined and ADI gene transcription was assayed by qRT-PCR. Letters indicate p-value < 0.05 in a two-way ANOVA with a Tukey’s multiple comparison test. *B*. *burgdorferi* (E) and *B*. *hermsii* (F) ADI protein expression levels were assayed by western blot in: (1) untreated, mid-logarithmic phase cells or cells grown with exogenously added (2) 0.5 mM L-arginine, (3) 1 mM L-arginine, or (4) 1 mM L-arginine and 0.5 mM L-ornithine combined, (5) empty, (6) recombinant ArcA_Bb_ or ArcB_Bb_. Primary antibody is indicated to right of each immunoblot panel and enolase is included as a loading control.

Previously, Dulebohn et al. showed that the addition of L-arginine to *B*. *burgdorferi* cultures abrogated the acid/proton stress effect caused by exposure to high levels of weak organic acids such as acetate and benzoate [55]. That study also found that *arc* (ADI) operon transcripts were upregulated with exposure to weak organic acids. In addition to contributing to acid/proton stress responses, L-arginine has been shown to promote growth and induce ADI operon expression in some bacterial species [13,55,56]. To investigate whether L-arginine promotes growth and activates ADI expression in *B*. *burgdorferi* and *B*. *hermsii* (S1 Table), both species were grown in BSK II with additional L-arginine alone or L-arginine and L-ornithine combined, added to the medium. In contrast to studies on other bacterial species, exogenously added L-arginine (> 5 mM) led to reduced growth in wild-type *B*. *burgdorferi* (BbWT) (p-value < 0.001) [10,15,57–59]. Surprisingly, the addition of L-ornithine to the culture medium reversed that effect (Fig 3A). Wild-type *B*. *hermsii* (BhWT), were able to tolerate exogenously added L-arginine up to 10 mM without a significant change in the growth rate (Fig 3B). The addition of L-arginine to mid-logarithmic BbWT cells, resulted in an increase in expression of each gene in the *arc* operon (p-value < 0.0001 for *arcA*_*Bb*_ and *arcB*_*Bb*_; p-value < 0.05 for *arcD*_*Bb*_) (Fig 3C and S2 Table). The addition of L-ornithine and L-arginine together removed the inhibitory effects of L-arginine alone and restored *arcA*_*Bb*_ (p-value < 0.0001) and *arcB*_*Bb*_ (P = 0.0009) transcripts to levels near those observed in untreated cultures (Fig 3C). In contrast, BhWT showed no statistically significant changes in gene expression of *arcABDC*_*Bh*_ when treated with L-arginine or L-ornithine (Fig 3D).

To assess whether differences in *arc* gene expression led to altered Arc protein levels, mid-logarithmic BbWT and BhWT grown with exogenously added L-arginine (0.5 mM or 1 mM) or in combination with L-ornithine (0.5 mM) were assayed for Arc protein expression (Fig 3E and 3F). In BbWT, ArcA_Bb_ and ArcB_Bb_ were not detected in cell lysates under any conditions tested (Fig 3E). In contrast, ArcA_Bh_ and ArcB_Bh_ were readily detected in BhWT in both treated and untreated cells but decreased slightly when the cells were grown in the presence of L-arginine alone, or L-arginine and L-ornithine combined (Fig 3F). The L-arginine/L-ornithine antiporter protein, ArcD, was detected in both species, and expression did not change under any of the conditions tested. To provide negative controls for western blots, we generated codon optimized expression vectors for *B*. *burgdorferi* ArcA_Bb_ and ArcB_Bb_, and *B*. *hermsii* ArcA_Bh_, ArcB_Bh_, and ArcC_Bh_ and purified each of these HIS-tagged ADI proteins (S1 Table). To show that the *B*. *hermsii* ArcA_Bh_ and ArcB_Bh_ polyclonal antibodies cross react with *B*. *burgdorferi* ADI proteins and to serve as a positive control, either rArcA_Bb_ or rArcB_Bb_, respectively, was loaded onto an outlying lane on each relevant gel (Fig 3E and 3F). To ensure that all protein signals were detected, *B*. *burgdorferi* western blots were developed with longer exposure times than *B*. *hermsii* western blots. Because of this, the control proteins are brighter in the *B*. *burgdorferi* ArcA_Bb_ and ArcB_Bb_ blots compared to the same *B*. *hermsii* blots, which required much less developing time to visualize the bands. Although this control did confirm that the antibodies reacted with the *B*. *burgdorferi* proteins in question, they did not directly quantify differences in the antibody reactivity. To test for differences in the antibody cross-reactivity to the different species’ ADI proteins, we prepared serial dilutions of recombinant ArcA_Bb_ and ArcA_Bh_ and ArcB_Bb_ and ArcB_Bh_ to directly compare their reactivity to the antibodies used in the study (S6 Fig). Results showed that ADI antibodies could detect *B*. *burgdorferi* ArcA and ArcB and showed similar reactivity to *B*. *hermsii* ArcA and ArcB.

These experiments highlight important differences in the regulation of the ADI in *B*. *burgdorferi* compared to *B*. *hermsii*. Experiments with *B*. *burgdorferi* showed that ADI transcripts were responsive to L-arginine and L-ornithine concentrations but were constitutively expressed in *B*. *hermsii*. Further, *B*. *burgdorferi* ArcA_Bb_ and ArcB_Bb_ expression levels were below the detection limit of these assays while *B*. *hermsii* ADI proteins were readily detected by western blot. Together these results may indicate that *B*. *burgdorferi* post-transcriptionally regulates both *arcA*_Bb_ and *arcB*_Bb_ to control protein levels while *B*. *hermsii* does not. The observed differences in ADI operon composition and sequence diversity, combined with differences in the response to extracellular L-arginine and L-ornithine concentrations suggest fundamental differences in the utilization of this important secondary metabolic pathway in these two spirochetal species.

### L-arginine utilization requires arginine deiminase expression

To further investigate the role of the ADI in spirochetal growth and L-arginine utilization, arginine deiminase (*arcA*) mutants were constructed in *B*. *burgdorferi* and *B*. *hermsii* as described in the Methods section and outlined in S5 Fig. In addition, we investigated the contribution of carbamate kinase, ArcC_*Bh*_ (*bh0843A*) to spirochetal growth and L-arginine utilization by generating a deletion mutant in BhWT (denoted as—BhΔ*arcC*) and a knock-in mutant in BbWT (denoted as Bb+*arcC*_Bh_) (S5 Fig). In contrast to studies on other bacterial species, growth curves showed there were no significant differences between WT and any of the ADI mutated strains (S7A and S7B Fig) [10,60]. Initially, we hypothesized that the heterologous expression of ArcC_*Bh*_ in *B*. *burgdorferi* might alter the expression patterns of the *arc* operon and provide the metabolic cue to relieve repression of ArcA_*Bb*_ and ArcB_*Bb*_. Although the expression of ArcC_*Bh*_ was readily detected in Bb+*arcC*_Bh_ lysates at levels similar to BhWT, its expression did not change the growth rate or affect the levels of ArcA_Bb_ or ArcB_Bb_ in *B*. *burgdorferi* (S7A and S7C Fig).

**Fig 4:**
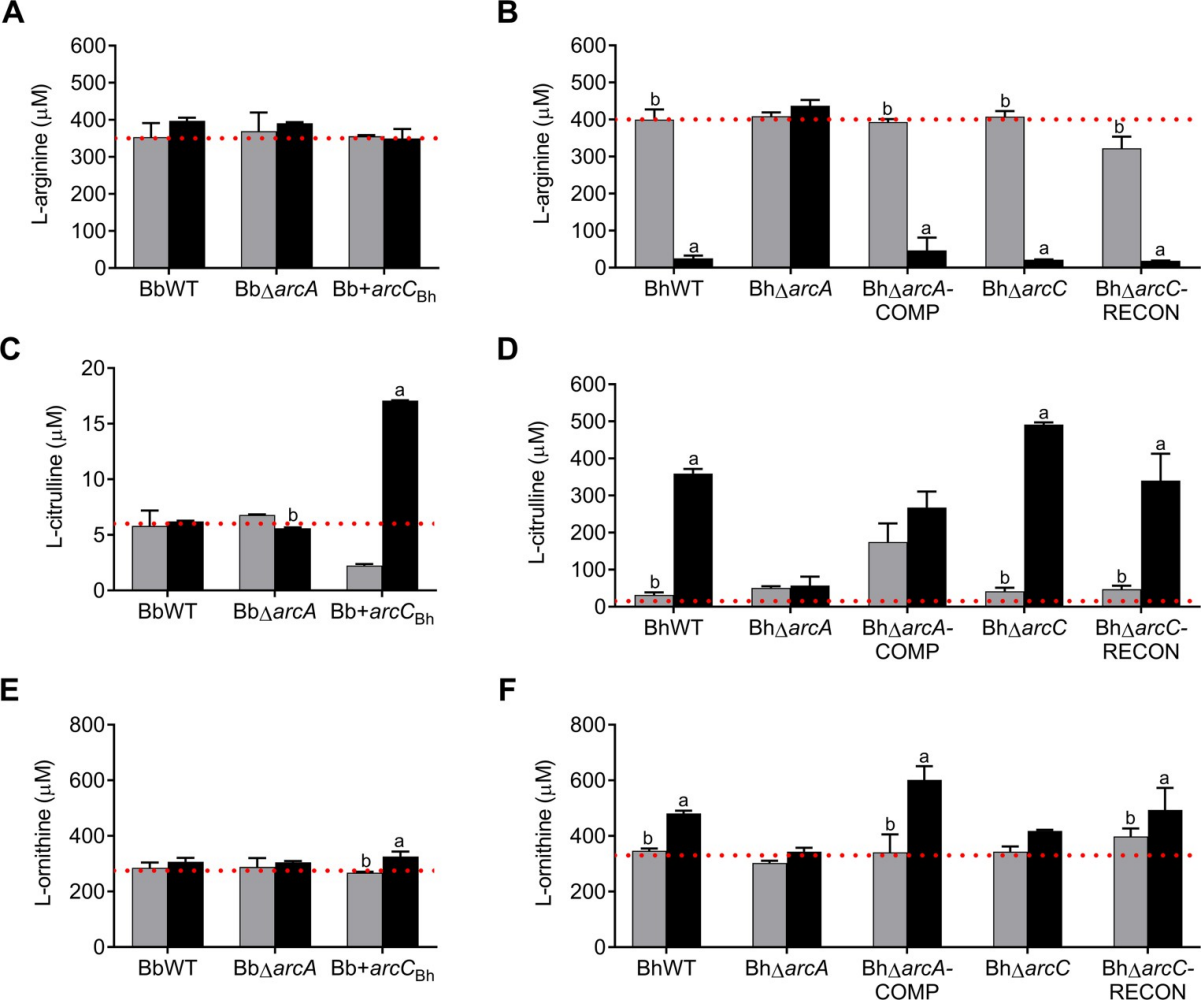
L-arginine utilization requires arginine deiminase expression during in vitro growth. *B*. *burgdorferi* (A, C, and E), *B*. *hermsii* (B, D, and F), and ADI mutant strains were grown to 5 x10^7^ (gray bars represent mid-logarithmic phase) and >1 x 10^8^ (black bars represent stationary phase), and supernatants from each culture were assayed for metabolite consumption and/or appearance. *B*. *burgdorferi* was grown in BSK II and *B*. *hermsii* in BSK X. Dotted line represents amino acid concentration in uninoculated growth medium (n = 3). Letters indicate statistical significance p-value < 0.05 in a two-way ANOVA with Dunnett’s multiple comparison test: (a) mid-logarithmic phase, (b) stationary phase.

To examine the role of the ADI in L-arginine, L-citrulline and/or L-ornithine utilization, supernatants were collected from cultures of each strain at cell densities corresponding to mid-logarithmic phase (5 x 10^7^) and late stationary phase (ca. 1–2 x 10^8^), and amino acid concentrations were assayed by high pressure liquid chromatography. In growth curve experiments (n = 3), none of the *B*. *burgdorferi* strains reduced the extracellular concentration of L-arginine, L-citrulline or L-ornithine (Fig 4A, 4C and 4E, respectively). Also, extracellular L-citrulline and L-ornithine did not accumulate as would be expected in the culture supernatant of a bacterium with a functional ADI (Fig 4C and 4E) [60]. In contrast, wild-type *B*. *hermsii* utilized L-arginine and excreted L-citrulline and L-ornithine in a growth phase/cell density dependent manner similar to observations in other bacteria (Fig 4B, 4D and 4F, respectively). In BhWT supernatants (mid-logarithmic phase compared to stationary phase), L-arginine levels decreased from 400.0 μM ± 27.5 μM to 25.1 μM ± 7.8 μM (p < 0.0001), L-citrulline levels increased from 31.5 μM ± 7.3 μM to 359.2 μM ± 12.8 μM (p < 0.0001), and L-ornithine levels increased from 347.2 ± 7.7 μM to 481.2 ± 9.9 μM (p = 0.0081). Supernatants taken from BhΔ*arcA* cultures showed that L-arginine disappearance was completely abrogated when arginine deiminase was deleted (Fig 3B). Complementation of *arcA*_Bh_, in BhΔ*arcA*-COMP, restored L-arginine uptake as well as L-citrulline and L-ornithine accumulation, at levels comparable to wild-type (Fig 4B, 4D and 4F, respectively). Neither the deletion of *arcC* (BhΔ*arcC*), nor its genetic reconstitution on the chromosome (BhΔ*arcC*-RECON) significantly affected the utilization of L-arginine or the extracellular accumulation of L-citrulline and L-ornithine in vitro (Fig 4B, 4D and 4F, respectively).

In uninoculated growth media and in supernatants taken from mid-logarithmic cultures, L-citrulline levels were quite low compared to L-arginine and L-ornithine concentrations (6.0 ± 0.9 μM versus 366.7 ± 19.7 and 285.1 ± 19.4, respectively) (Fig 4). While the addition of *arcC*_Bh_ to the *B*. *burgdorferi* genome did not affect growth or detection of ArcA_Bb_ or ArcB_Bb_, heterologous expression of carbamate kinase did affect the production L-of citrulline and L-ornithine. Supernatants from Bb+*arcC*_Bh_ showed a statistically significant increase in L-citrulline and L-ornithine appearance (up to 17 μM ± 0.2, p < 0.0001 and 325.4 ± 12.7, p < 0.0155, respectively) at stationary phase compared to both BbWT (ns) and BbΔ*arcA* (ns) (Fig 4). These data suggest that although the ArcA_Bb_ and ArcB_Bb_ proteins were below our limit of detection, there is likely low levels of activity and the addition of carbamate kinase modestly increased the turnover of L-arginine to L-citrulline and then L-ornithine. While the observed increase in L-citrulline and L-ornithine in Bb+*arcC*_Bh_ was statistically significant, the amounts produced were dramatically less than that produced by BhWT during stationary phase.

These experiments showed that while ArcA and ArcC were not required for growth, expression of the ADI enzymes affected L-arginine catabolism (S7A and S7B Fig) and further showed that arginine deiminase expression is indispensable for the direct utilization of L-arginine from the environment in *B*. *hermsii* (Fig 4).

### Infection with *B*. *burgdorferi* leads to increased plasma L-ornithine levels in the murine host

**Fig 5:**
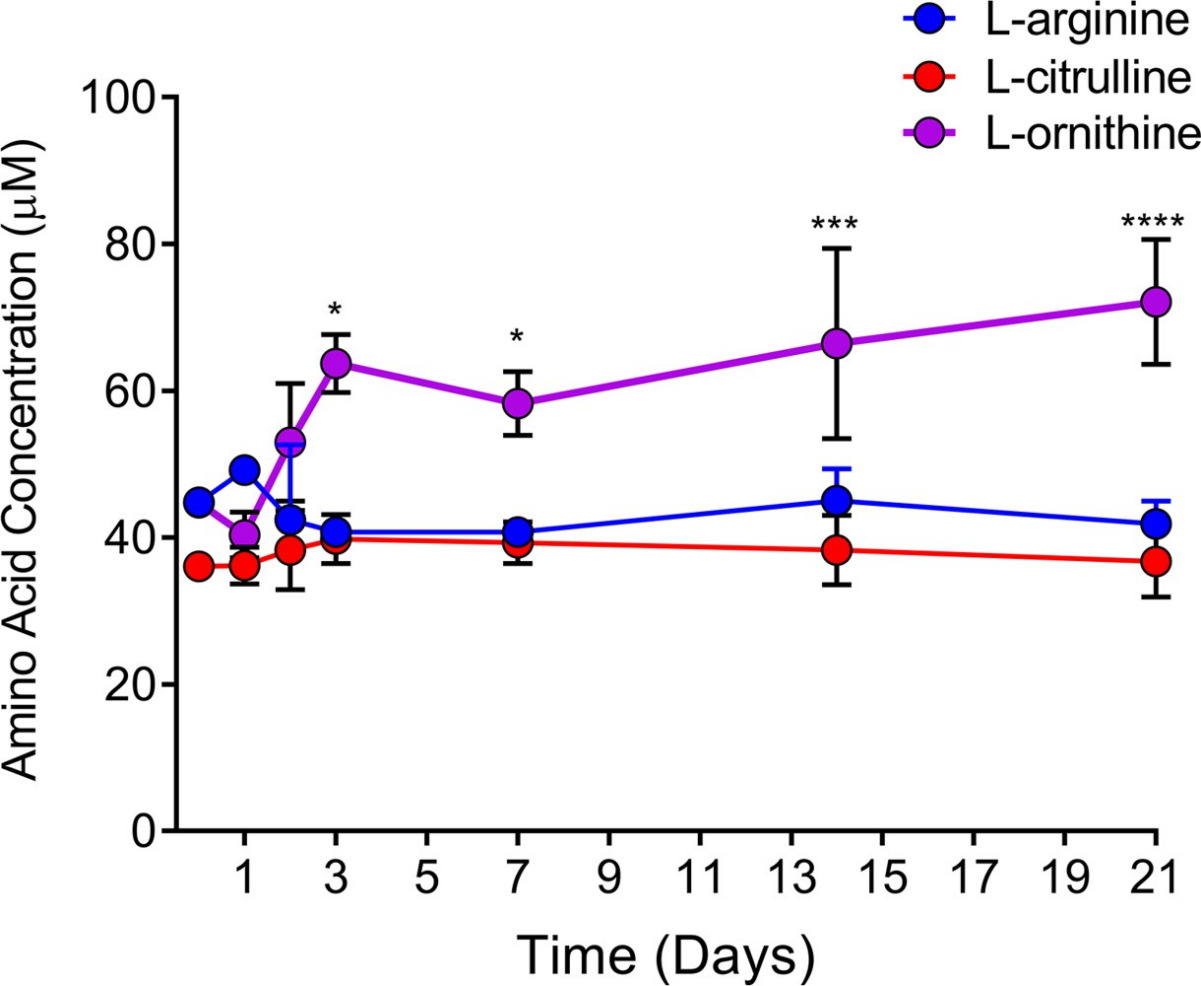
L-ornithine, but not L-arginine or L-citrulline plasma levels become elevated during *B*. *burgdorferi* infection. Outbred Swiss webster mice were infected with wild-type *B*. *burgdorferi* and plasma concentrations of L-arginine, L-citrulline, and L-ornithine were quantified by HPLC analysis. Individual, one-way ANOVAs with Tukey’s multiple comparison test were performed to evaluate significant changes in amino acid concentration over time. Asterisks indicate p-value ≤ 0.05.

To test whether *B*. *burgdorferi* infection affected plasma L-arginine, L-citrulline, or L-ornithine concentrations, a cohort of 35 mice were infected with 5 x 10^4^ spirochetes (BbWT), five mice were euthanized at each time point (0, 1d, 2d, 3d, 7d, 14d, 21d) and plasma amino acid levels were quantified. Consistent with the results observed during in vitro experiments, neither L-arginine nor L-citrulline plasma levels significantly changed at any of the timepoints examined throughout the experiment (Fig 5). However, over the course of the infection L-ornithine concentration increased significantly in the plasma of BbWT infected mice, becoming elevated on day three compared to time zero (p = 0.0101), and day 1 (p = 0.0008). L-ornithine levels remained elevated through day 21, (day 7, p = 0.0176; day 14, p = 0.0002; day 21, p < 0.0001), compared to time zero (Fig 5). Although in vitro growth experiments with *B*. *burgdorferi* showed that L-ornithine levels were not significantly altered in the growth medium, (Fig 4) the increase in plasma L-ornithine was surprising and may be related to the upregulation of polyamine synthesis in the host [61].

### Neither arginine deiminase nor carbamate kinase were required for infection of mice or ticks

**Fig 6:**
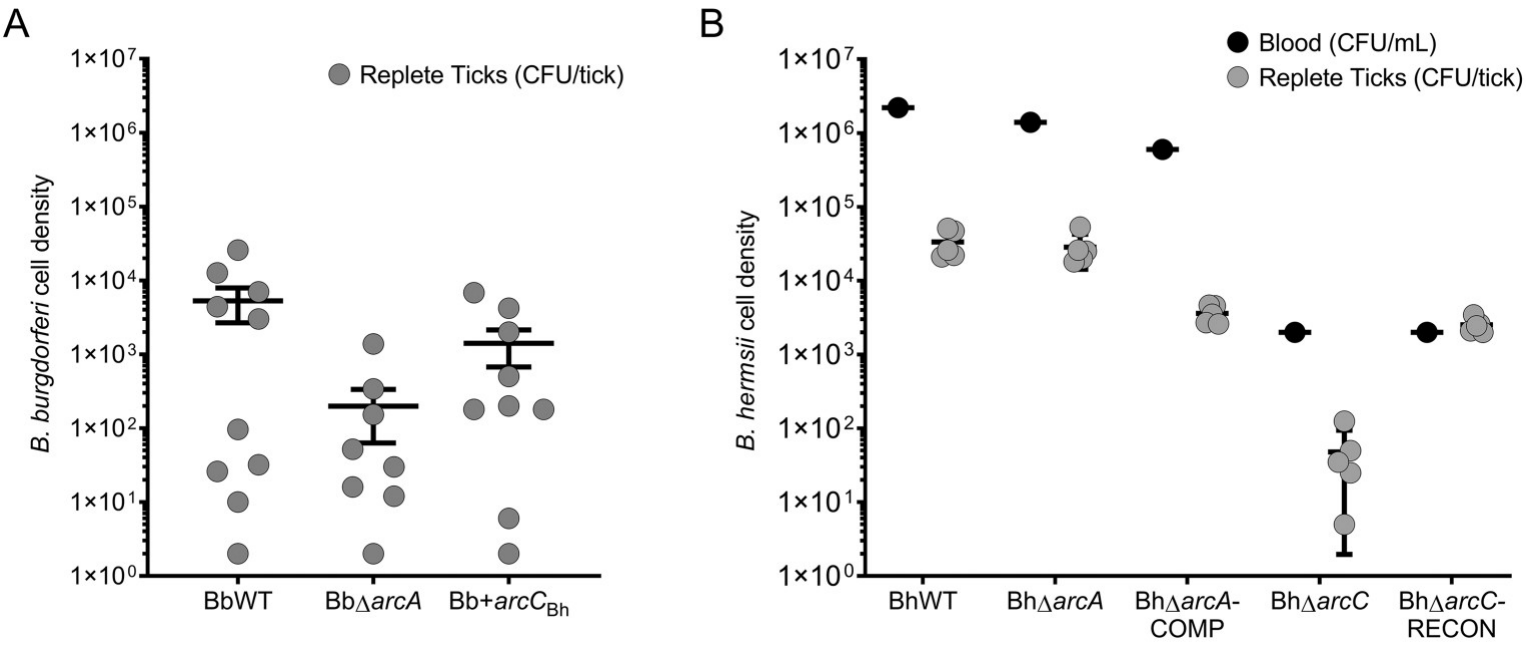
Acquisition of *B*. *burgdorferi* and *B*. *hermsii arc* mutants by their respective tick vectors. A) Uninfected, larval *I*. *scapularis* ticks fed on mice infected with *B*. *burgdorferi* (either BbWT, BbΔ*arcA* or Bb+*arcC*_Bh_) and upon repletion individual ticks were crushed and plated, and spirochetes were enumerated. B) Uninfected, nymphal *O*. *hermsi* ticks fed on spirochetemic mice infected with *B*. *hermsii* (wild-type or *arc* mutants) and spirochetes were enumerated by plating infected blood and crushed *O*. *hermsi* within 24 h of ticks dropping off mice.

To investigate the role of the ADI in *B*. *burgdorferi* infectivity, 10 naïve mice were infected intraperitoneally (IP) with each individual *B*. *burgdorferi* ADI mutant (BbWT, BbΔ*arcA*, or Bb+*arcC*_Bh_). Mice were assayed for infection by culturing multiple tissues (tibiotarsus joint (5/5), bladder (5/5), and skin (5/5)) in BSK II growth medium. Experiments showed that each tissue assayed was readily colonized, resulting in a 100% infection rate for each of the strains tested. To determine that *B*. *burgdorferi* ADI mutants could be acquired by *I*. *scapularis* during feeding, a subsequent experiment was conducted. Two additional mice were inoculated with each *B*. *burgdorferi* ADI mutant strain and upon confirmation of a positive infection by culture of an ear punch biopsy, 50 larval *I*. *scapularis* ticks were fed upon each mouse. Analysis of the spirochete burden showed that *I*. *scapularis* ticks were able to acquire BbWT (10/10), BbΔ*arcA* (8/10) or Bb+*arcC*_Bh_ (9/10) at a similar frequency (Fig 6A). Although there were fewer spirochetes, on average, acquired by ticks fed on mice infected with BbΔ*arcA*, the difference was not statistically significant (Fig 6A). To assess whether arginine deiminase played a role in transmission from the tick to the mammalian host, 5 infected ticks (WT or BbΔ*arcA*) were placed on naïve mice (n = 6) and allowed to feed to repletion. Tick feeding experiments showed that 100% of mice were infected and further showed that arginine deiminase is not required for the completion of the tick-mouse infectious cycle in *B*. *burgdorferi*.

To assess *B*. *hermsii arc* mutants for infectivity in mice and ticks, mice were infected with each strain (BhWT, BhΔ*arcA*, BhΔ*arcA*-COMP, BhΔ*arcC*, BhΔ*arcC*-RECON) at a dose of 1 x 10^3^ spirochetes/mouse and the blood was monitored for spirochetes daily by microscopy for up to seven days. The parental wild-type strain and all BhΔ*arc* strains were able to infect mice and successfully relapse resulting in a 100% infection rate (10/10 mice per strain). These results showed that arginine deiminase and carbamate kinase are dispensible for *B*. *hermsii* to cause a relapsing infection. To determine if deletion of *arcA*_Bh_ or *arcC*_Bh_ affected acquisition of the spirochetes by their tick vector *O*. *hermsi* ticks were fed on mice infected with BhWT, and each ADI mutant (Fig 6B). *O*. *hermsi* is a fast feeding, soft bodied ticks that require the host to have an active bacteremia to acquire the spirochetes [33,62]. Upon confirmation of spirochetemia with each strain, *O*. *hermsi* ticks were fed upon each infected mouse and an aliquot of blood was plated to enumerate the number of spirochetes in the blood during tick feeding. Additionally, 4–6 *O*. *hermsi* ticks were crushed and plated immediately following drop-off (Fig 6B). Due to individual variation in levels of spirochetemia, we observed different levels of bacteremia in each infected mouse at the time of tick feeding. This accordingly affected the number of spirochetes that *O*. *hermsi* ticks were able to acquire, with BhΔ*arcC* and BhΔ*arcC*-RECON having similarly lower spirochete levels in both the blood and infected ticks at the time of the tick feeding. Despite these individual differences, *O*. *hermsi* nymphs successfully acquired each strain from spirochetemic mice, showing that neither arginine deiminase nor carbamate kinase were required for acquisition by *O*. *hermsi* ticks. Interestingly, plating results showed that ticks feeding on mice infected with BhΔ*arcC* had reduced CFUs compared to ticks feeding on mice infected with BhΔ*arcC*-RECON, suggesting a potential role for carbamate kinase in *Ornithodoros* colonization.

Although there were differences between BbWT and BhWT in growth and gene transcription patterns in response to L-arginine as well as in utilization of L-arginine from the environment, neither species was hindered from infecting mammals or ticks when the ADI was genetically mutated. These results differ from observations in other bacteria, where deletion of arginine deiminase and/or carbamate kinase led to loss of virulence [10,13].

### Arginine deiminase promotes bacterial survival during mammalian infection with *B*. *hermsii*

**Fig 7:**
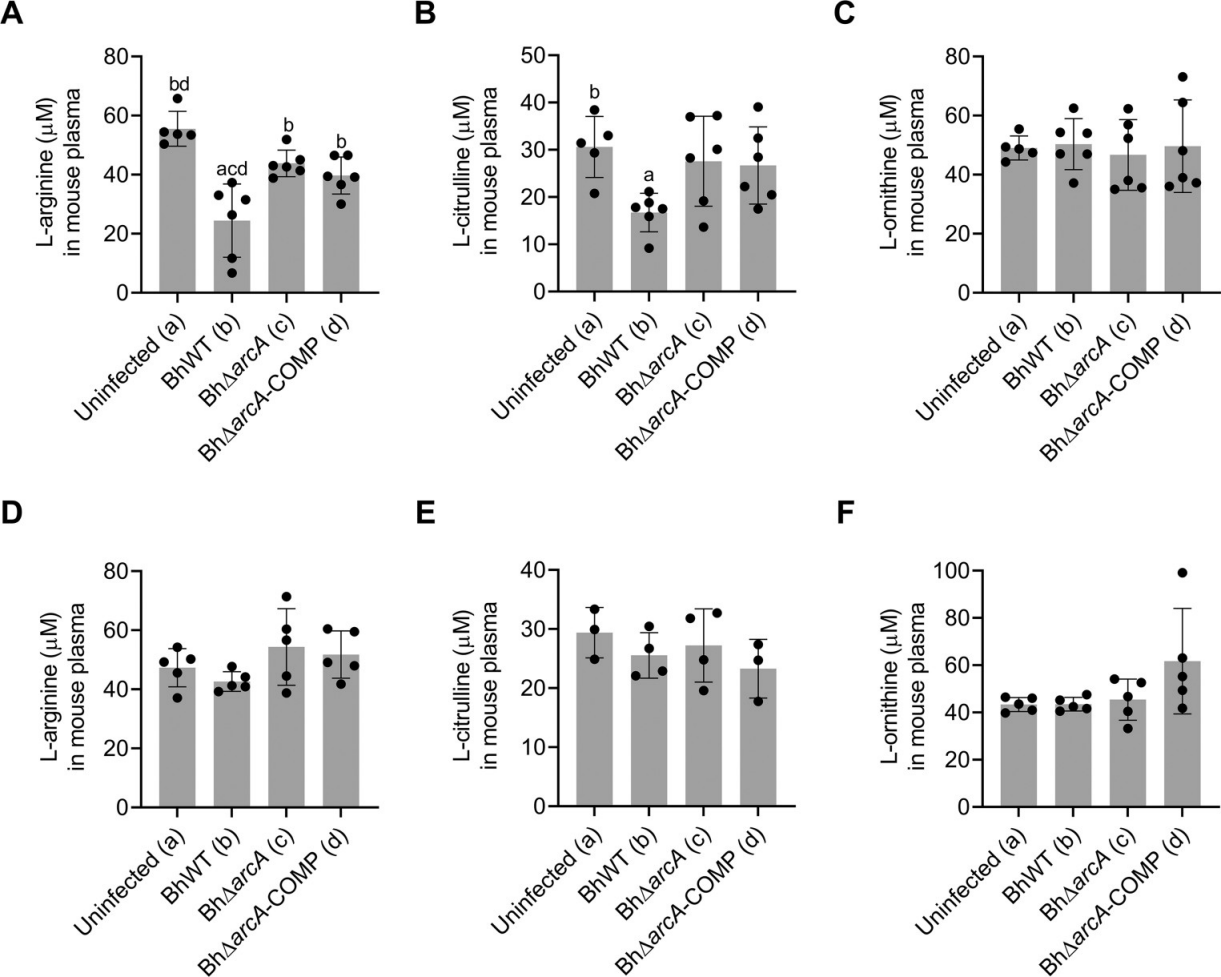
L-arginine depletion is dependent on ArcA during *B*. *hermsii* infection in mice. Mice were inoculated with BhWT, BhΔ*arcA*, or BhΔ*arcA*-COMP by IP injection and cohorts were euthanized at day 5 (A-C; n = 6) and day 15 (D-F; n = 5). L-arginine (A and D), L-citrulline (B and E), and L-ornithine (D and F) concentrations in mouse plasma were quantified by HPLC analysis as described in the materials and methods. Letters denote p-value < 0.05 in a one-way ANOVA with a Tukey’s multiple comparison test.

Although arginine deiminase was not required for infection, we sought to determine whether arginine deiminase promotes survival and plays a role in L-arginine utilization during mammalian infection. To do this, the mouse plasma was assayed for L-arginine, L-citrulline, and L-ornithine concentrations by HPLC analysis on days 5 and 15 (Fig 7). Mice infected with BhWT showed significantly reduced levels of L-arginine (p < 0.0001) and L-citrulline (p = 0.0369) in their plasma at day 5 but not day 15 (P.I.) (Fig 7A, 7B, 7D and 7E). In contrast, BhΔ*arcA* infected mice showed only a modest decrease in plasma L-arginine levels compared to uninfected mice (day 5, p = 0.3556) (Fig 7A). Mice infected with BhΔ*arcA*-COMP, had reduced plasma L-arginine at levels similar to wild-type infected mice (p = 0.0132) (Fig 7A). While BhWT infected mice had consistently lower levels of L-citrulline, the levels of L-citrulline and L-ornithine varied substantially between individual mice. Although mice infected with BhΔ*arcA*-COMP did show reduced L-arginine levels, those mice did not have reduced citrulline levels (Fig 7B).

**Fig 8:**
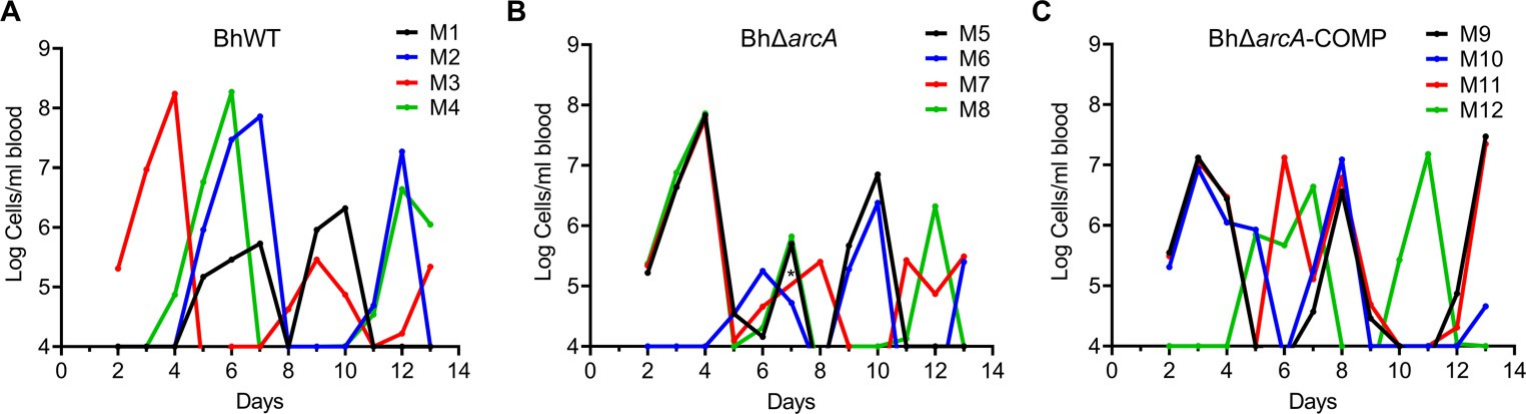
Relapse profile of BhWT, BhΔ*arcA* and BhΔ*arcA*-COMP. Mice were infected by intraperitoneal needle inoculation with 1 x 10^3^ spirochetes of A) BhWT, B) BhΔ*arcA*, and C) BhΔ*arcA*-COMP. Spirochete numbers in blood were enumerated every 24 h beginning at day 2 post inoculation using quantitative PCR. M1-M12 indicate individual mice used in infection study. Asterisk in panel B indicates sample was not collected for this time point (day 7, M7).

**Fig 9:**
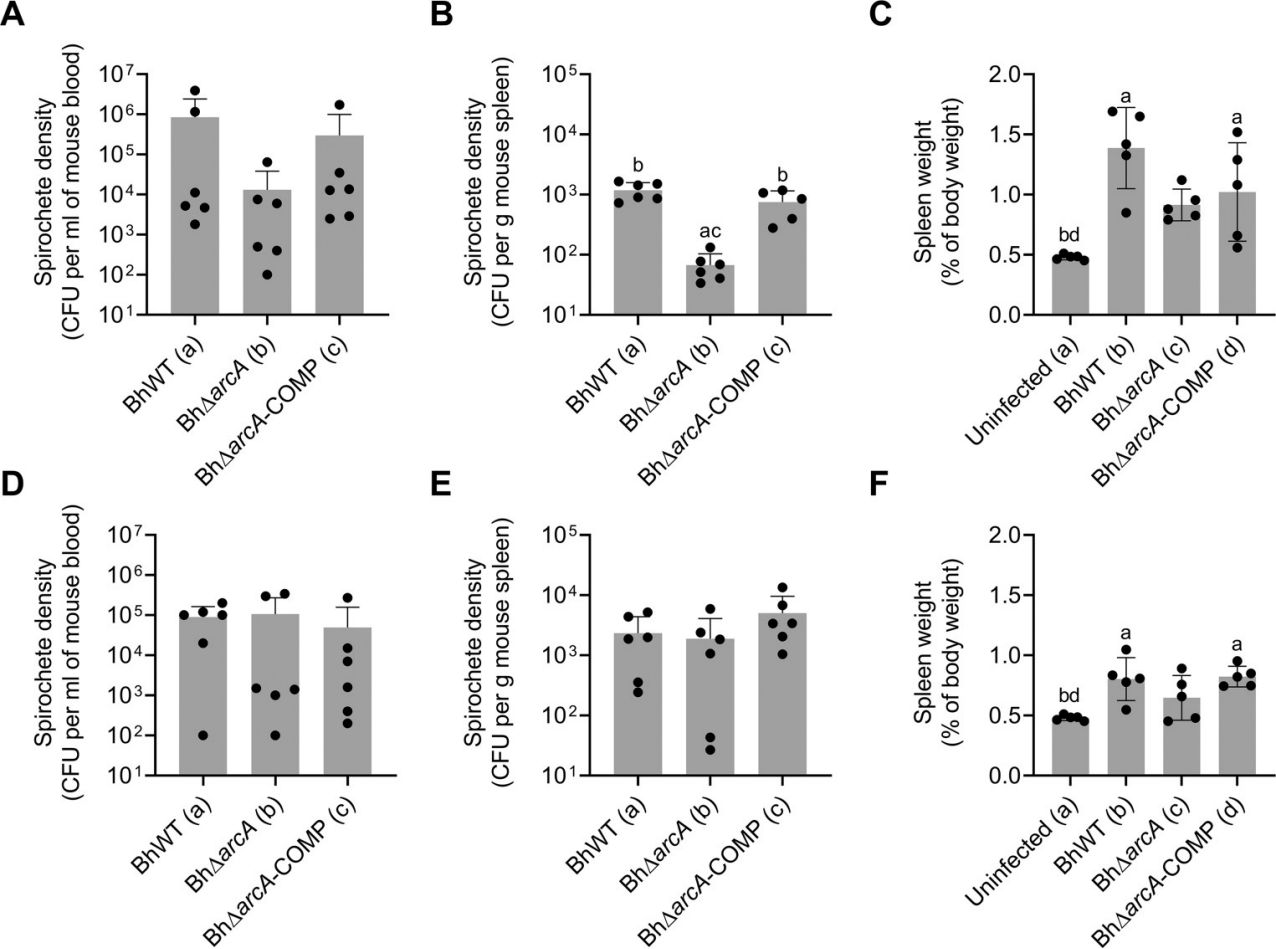
Arginine deiminase contributes to the survival of *B*. *hermsii* in the spleens of infected mice. Mice were inoculated with BhWT, BhΔ*arcA*, or BhΔ*arcA*-COMP by IP injection and cohorts were euthanized at day 5 (A-C; n = 6) and day 15 (D-F; n = 5). Mouse blood (A and D) and spleens (B and E) were plated to determine the number of spirochetes. Prior to euthanasia, mice were weighed, and spleens were excised and immediately weighed (C and F). Letters indicate P-value < 0.05 in a one-way ANOVA Tukey’s multiple comparison test.

Due to the requirement for arginine deiminase expression to utilize L-arginine for growth and energy production, we hypothesized that BhΔ*arcA* would show reduced bacterial densities and/or fewer bacteremic relapses during murine infection. To test this hypothesis, mice were infected with either BhWT, BhΔ*arcA*, or BhΔ*arcA*-COMP and spirochete genome copies were quantified by qPCR in blood samples taken daily, for 13 days, from each mouse (Fig 8). Although there were differences in the relapse profiles of the individual mice, the arginine deiminase mutant strain and its complement were able to readily infect mice, causing multiple relapses and reaching similar initial densities to wild type. To further quantify bacterial survival during infection and determine whether cells detected by qPCR were viable, whole blood and homogenized spleen samples were collected and plated to quantify viable spirochetes on days 5 and 15 (Fig 9). In addition, to measure differences in splenomegaly characteristics, mice were weighed and following excision, spleens were also weighed to yield spleen weights as a percentage of total body weight. While each *B*. *hermsii* strain reliably infected the murine host, mice infected with BhΔ*arcA* had lower numbers of viable bacteria (compared to BhWT) in the blood (ns, P = 0.3383) and spleen (P < 0.0001), at day 5 but not day 15 (P. I.) (Fig 9A and 9B, respectively). In addition, mice infected with BhWT had a significant increase in splenomegaly development compared with mice infected with BhΔ*arcA*, during the early stage of the infection. It is important to note that mice infected with the BhΔ*arcA*-COMP did not reduce L-arginine and L-citrulline plasma levels to the same extent as wild-type (Fig 7) and showed more individual variation in the relapse kinetics (Fig 8). However, culturing experiments clearly showed that complementation of arginine deiminase restored the ability of BhΔ*arcA*-COMP to survive in the spleen of infected animals. These experiments suggest that the ability to utilize L-arginine by BhWT confers an advantage during the early phase of the infection, leads to improved growth and survival in the blood and spleen. Unlike in vitro assays, where L-citrulline and L-ornithine accumulated in the growth media concurrent with the disappearance of L-arginine, in vivo infection experiments showed systemic depletion of L-arginine and L-citrulline levels in BhWT infected mice while L-ornithine levels remained stable.

## Discussion

It has been shown that ADIs can play multiple roles in bacterial survival, and, in some cases, they are important for virulence [9,10,24,63]. For example, in a murine model of inflammatory infection of cutaneous tissue, Δ*arcA* and Δ*arcB* mutants in *Streptococcus pyogenes* were shown to be attenuated and hyper-attenuated, respectively [10]. The ADIs have been shown to contribute to the virulence of both *Salmonella* and *Listeria* during gastrointestinal infections and to *Porphyromonas gingivalis* biofilm formation, which is essential for gingival colonization [64,65]. It has been observed that the composition, complexity, and regulation of bacterial ADIs are adapted to fit each species environmental niche and can be upregulated to supplement growth and/or combat specific stress conditions [24,46,56,60,65–68]. In this study, analyses of the respective ADIs revealed unique adaptations in *B*. *burgdorferi* and *B*. *hermsii* including differences in growth, gene and protein expression and L-arginine utilization during mammalian infection. Although arginine deiminase was dispensable for mammalian and tick acquisition in both species, experiments with *B*. *hermsii* showed that the recovery of viable bacteria from the blood and spleen during acute infection was dramatically decreased by deletion of *arcA* (encoding arginine deiminase) (Fig 9).

In general, the genes that compose bacterial ADIs are organized in a single operon; however, significant variation in the composition and complexity of the *arc* operon has been observed (Fig 2) [50,56,67,69,70]. ADIs are tightly regulated in bacteria and can be affected by carbon levels (carbon catabolite repression), anaerobiosis, growth phase, pH, and extracellular L-arginine levels. In some bacterial species, ADI regulation is additionally controlled by transcriptional activation and/or repression through the transcriptional regulator ArgR; however, none of the *Borreliella* or *Borrelia* species examined in this study contain an ArgR homologue [56,64,67,68,71]. Phylogenetic analysis of the *arc* genes showed clustering of each gene consistent with current phylogenetic delineations among *Borreliella* and *Borrelia* species, with LD and RF spirochetes consistently clustering into separate groups (S1–S4 Figs). A previous study on the evolution of ADIs found that the *arc* genes found in *Borreliella* and *Borrelia* were likely the result of horizontal transfer from a low G + C containing prokaryotic ancestor [51]. Our analyses further showed that ADI genes have diverged among the LD and RF spirochetes and suggest that individual genes may be experiencing more selective pressures leading to sequence divergence over time.

In addition to differences in ADI operon sequence and composition, differences were observed in growth and gene transcription when cells were exposed to exogenously added L-arginine. *B*. *burgdorferi* upregulated *arc* genes in response to increasing extracellular L-arginine concentrations and that increase in transcription was abrogated by the addition of L-ornithine (Fig 3C). In a pattern distinct from *B*. *burgdorferi*, *B*. *hermsii* ADI gene expression patterns did not significantly change in the presence of extracellular L-arginine or L-ornithine suggesting a constitutively active ADI operon (Fig 3D). *B*. *burgdorferi* and *B*. *hermsii* had significantly different ADI protein expression patterns as well. *B*. *burgdorferi* ArcA and ArcB proteins were not detected by immunoblot during in vitro growth, while *B*. *hermsii* ADI proteins were readily detected (Fig 3E and 3F). Additionally, *B*. *burgdorferi* growth was inhibited in the presence of increasing levels of L-arginine, an effect that was not observed in *B*. *hermsii* growth experiments (Fig 3A and 3B). The addition of L-ornithine to *B*. *burgdorferi* cultures at 1/20 the equimolar concentration of L-arginine ameliorated the L-arginine-stimulated growth defect, suggesting that *B*. *burgdorferi* is exquisitely sensitive to the levels of L-arginine and L-ornithine in its environment. This was unique to *B*. *burgdorferi* and is likely due to differences in the action of the antiporter, ArcD, which canonically exchanges L-arginine (in) for L-ornithine (out) in response to the intracellular and extracellular concentrations of those metabolites [50]. Further work will be required to determine the factors that contribute to the tight ADI regulation observed in *B*. *burgdorferi* uncovered in this study.

Because in vitro experiments showed that *B*. *burgdorferi* did not utilize L-arginine directly, it was not surprising that wild-type infected mice did not have reduced plasma levels of L-arginine. However, the observed increase in plasma L-ornithine was unexpected. In mammals, the enzyme arginase II generates L-ornithine and is the first step in polyamine production by phagocytic cells (alternative activation pathway in macrophages) [72]. In a murine Lyme arthritis and carditis model, Lasky et al. showed that alternatively activated macrophages expressing arginase II outnumbered classically activated macrophages 100-fold throughout their six-week infection experiment [61). In our study, L-ornithine was readily available in mouse plasma and levels significantly increased during a wild-type *B*. *burgdorferi* infection. In general, macrophage polarization can dramatically affect infection outcomes and the expression of host arginases to produce polyamines is considered an anti-inflammatory signal. While our data are consistent with Lasky et al. [61], the role for L-ornithine and other polyamines in *B*. *burgdorferi* colonization and persistence in mammalian hosts has not yet been elucidated. A recent study by Jutras et al. characterized the peptidoglycan of *B*. *burgdorferi*, confirming that L-ornithine is a structural component of its peptidoglycan and was the third most abundant amino acid identified behind D-alanine and L-glycine [73]. Because L-ornithine is present at each step of the infectious cycle, it is possible that *B*. *burgdorferi* evolved to repress ArcA and ArcB since L-ornithine can be scavenged directly from its environment and, lacking *arcC*, they do not derive energy from L-arginine metabolism [74–76]. It is also possible that *B*. *burgdorferi* can supplement the L-ornithine required for growth by utilizing L-arginine and/or L-ornithine derived from peptides imported via the oligopeptide permease (Opp) system [77]. This theory is consistent with the fact that several other Lyme disease spirochetes lack *arcD* and must rely on L-arginine and/or L-ornithine via peptide transport to generate peptidoglycan. To date, only acid/proton stress has been shown to positively regulate the ADI in *B*. *burgdorferi* [55]. More work will be required to identify the mechanisms of regulation of the ADI in *B*. *burgdorferi*.

In contrast to *B*. *burgdorferi*, *B*. *hermsii* reduced extracellular L-arginine levels, both in vitro and in vivo, in an arginine deiminase dependent manner (Figs 4 and 8). The lower levels of both L-arginine and L-citrulline in the plasma of mice infected with wild-type *B*. *hermsii*, indicated a general disruption in L-arginine homeostasis during the acute phase of the RF infection [78,79]. L-arginine is considered a nonessential amino acid in humans and mice i.e., it can be acquired through de novo synthesis, citrulline recycling, and protein turnover, but can become conditionally essential during infections [75,80]. In addition to being a critical substrate for polypeptide synthesis and energy production (TCA cycle) in eukaryotes, L-arginine is the sole substrate for nitric oxide synthase (iNOS) and arginase enzymes, which convert L-arginine into nitric oxide (NO) and L-ornithine, respectively. Although it has been observed that systemic L-arginine depletion can modulate the host immune system during multiple different disease states, it is currently unknown if utilization of L-arginine by *B*. *hermsii* affects host immune responses to promote infection [9,21,81–83]. Fewer numbers of viable bacteria were recovered from the blood and spleen of mice infected with BhΔ*arcA* compared to BhWT, during the early, acute phase of infection (day 5). The mammalian spleen is an important immunological site for host defenses and participates in antibody formation, lymphocyte and monocyte production and maturation, blood filtration, phagocytosis, and red blood cell homeostasis [84]. While the spleen is important for developing immune responses that facilitate bacterial clearance, particularly in blood-borne diseases, its role in promoting bacterial clearance during *B*. *hermsii* infection has remained largely uncharacterized. More investigations into how the utilization of L-arginine promotes *B*. *hermsii* growth in the blood and spleen of infected host mammals.

The experiments in this study revealed important differences in the ADIs of *B*. *burgdorferi* and *B*. *hermsii* and these differences have physiological consequences to both the bacteria and the hosts they infect. The observed sensitivity of *B*. *burgdorferi* to both arginine and ornithine concentrations, altering both growth and transcriptional activities in response, suggests an important role for L-arginine and L-ornithine during its lifecycle. More work will be required to identify the mechanisms of regulation of the ADI in *B*. *burgdorferi* as well as to characterize the role of host supplied L-ornithine in *B*. *burgdorferi* survival and persistence in mammals. In addition, these experiments have provided an initial characterization of an important ATP-generating metabolic pathway in *B*. *hermsii* and have demonstrated the role of arginine deiminase in L-arginine transport and catabolism during mammalian infection.

## Methods

### Ethics statement

Mouse studies were conducted at the Rocky Mountain Laboratories and all protocols approved by the Animal Care and Use Committee. The Rocky Mountain Laboratories are accredited by the International Association for Assessment and Accreditation of Laboratory Animal Care (AAALAC). All animal work was done according to protocols approved by the Rocky Mountain Laboratories Animal Care and Use Committee (Protocol Number 2017–002, 2018–061).

### Bacterial strains, culture and growth curve conditions, and reagents

*B*. *burgdorferi* strain B31-A3 [85] and derivatives were grown in Barbour-Stoenner-Kelly medium (BSKII) with 6% rabbit serum, while *B*. *hermsii* strain DAH [86] and derivatives were grown in BSKII with 12% rabbit serum (S1 Table). All liquid and plated cultures were incubated microaerophilically (5% CO_2_ and 3% O_2_) in a Forma Series II Water Jacket CO_2_/O_2_ incubator (Thermo Fisher Scientific, Inc., Waltham, MA, USA). For growth experiments in the presence of increasing levels of exogenously added L-arginine and/or ornithine, 1 M solutions of either L-arginine or ornithine were prepared in sterile water, filter sterilized and added to the growth medium at the indicated concentrations. Spirochetes were generally enumerated by dark-field microscopy. Specifically, 10 μL of cultures were placed on a glass slide (VWR, Radnor, PA) and a wet mount using a 22 x 22 mm coverslip was prepared for counting spirochetes. *Escherichia coli* was used for cloning and plasmid maintenance. Antibiotics were used at the following concentrations: chloramphenicol and gentamicin at 20 μg ml^-1^, streptomycin at 50 μg ml^-1^, and spectinomycin at 100 μg ml^-1^. All chemicals were purchased from Sigma-Aldrich (St Louis, MO) unless stated otherwise.

### Phylogenetic analysis of ADI sequences

Nucleotide sequences for arginine deiminase, ornithine transcarbamylase, carbamate kinase and the L-arginine/L-ornithine antiporter (*arcD*) were obtained from GenBank and ERGO [53]. Inferred amino acid sequences were aligned using MAFFT and Maximum Likelihood trees were inferred using RAxML available in MegAlign Pro (DNASTAR Lasergene 17) [87]. Statistical support for branches was assessed by multiparametric bootstrapping (1000 replicates). Bacterial species analyzed include *B*. *burgdorferi* B31-A3, *B*. *burgdorferi* JD1, *B*. *burgdorferi* Zs7, *B*. *mayonii* MN1420, *B*. *garinii* PBi, *B*. *afzelii* ACA-1, *B*. *spielmanii* A14s, *B*. *valaisiana* VS116, *B*. *crocidurae* str. Achema, *B*. *duttonii* Ly, *B*. *recurrentis* A1, *B*. *coriaceae* Co53, *B*. *turicatae* BTE5EL, *B*. *parkeri* SLO, *B*. *hermsii* MTW, *B*. *hermsii* DAH, *B*. *hermsii* HS1, *B*. *anserina*, *B*. *miyamotoii* LB-2001, *Lactobacillus brevis* ATCC 367, *Pseudomonas aeruginosa* PAO1, *Salmonella enterica* subsp. *Typhimurium* LT2, and *Listeria monocytogenes* EGDe, and *Staphylococcus aureus* NCTC 8325.

### Generation of ADI pathway mutants and complemented strains

To generate an *arcA* deletion mutant in *B*. *burgdorferi*, the suicide vector pCR102 (pPCR-script Cam::Δ*arcA*::*aacC1*) was created for allelic exchange. To do this, approximately 0.6 kB of DNA 5’ and 3’ to *bb0841* were PCR amplified using primers P1 & P2 and P3 & P4 (S2 Table), respectively. The 5’ region, the antibiotic resistance cassette, and the 3’ region of *bb0841* were sequentially cloned into pPCR-script CAM SK(+) (Agilent Technologies, Cedar Creek, TX). The *aacC1* gene including the *flgB*_Bb_ promoter, which confers gentamicin resistance, was PCR amplified using primers P5 & P6 from pBSV2G (S1 and S2 Tables) [88]. Wild-type *B*. *burgdorferi* B31-A3 (BbWT) were transformed with pCR102 as previously described and transformants were selected by plating with 20 μg ml^-1^ gentamicin and 500 μM ornithine monohydrochloride (Sigma) [89]. The location and orientation of the inserted gentamicin cassette was confirmed using primers P7 & P8 and P9 & P10. One clone was confirmed to be isogenic to the parent strain and was named BbΔ*arcA*. Numerous attempts to complement *bb0841* were unsuccessful and included attempts to reintroduce the orf into its original location as well as at the 3’ end of the *bb0843* orf.

To generate an *arcA* deletion mutant in *B*. *hermsii*, a PCR fragment was amplified from wild-type *B*. *hermsii* genomic DNA containing approximately 1 kB of DNA 5’ and 3’ to *bh0841* using primers P11 & P12 and was cloned into pCR-XL-TOPO (Thermo Fisher Scientific, Inc.). The plasmid was linearized with NdeI and the *bh0841* ORF was eliminated by PCR using primers P13 & 14. The *aadA* gene was amplified from pKFSS1 [90], which confers streptomycin resistance, using primers P15 & P16. The *aadA* was cloned into the amplified plasmid by Gibson Assembly (New England Biolabs Inc., Ipswich, MA) to generate the suicide vector pSS100 (pCR-XL-TOPO::Δ*arcA*::*aadA*). Wild-type *B*. *hermsii* DAH (BhWT) was transformed with approximately 25 μg DNA and plated as previously described [91]. Transformants were selected by plating on 50 μg ml^-1^ streptomycin and 500 μM ornithine monohydrochloride (Sigma). Cassette insertion and orientation was confirmed by PCR with primers P51 & P55 and P52 & P56, and the clone was named BhΔ*arcA*. To complement *arcA* in BhΔarcA, *bh0841* and its putative native promoter (200 bp upstream) were reintroduced at the end of the *arc* operon, 3’ to *bh0843A*. To generate BhΔ*arcA*-COMP, a suicide vector was constructed, pSS101 (pzero-blunt TOPO::*arcA*_Bh_-*flgBp*::kan^R^), and was introduced to BhΔ*arcA* by electroporation. To generate pSS101, approximately 1 kB of DNA 5’ and 1.5 kB 3’ to bh0843A was PCR amplified using primers P17 & P18 and cloned into the Zero Blunt TOPO vector (Thermo Fisher Scientific, Inc.). This plasmid was linearized with BsgI and amplified with primers P23 & P24. *arcA*_Bh_ (*bh0841*) plus 200 bp 5’ to the start codon were PCR amplified using primers P19 & P20 and was cloned into a plasmid containing a kanamycin resistance cassette, pTA-flgBpKan [92] with restriction enzymes EcoRV and XhoI. The *bh0841* orf and promoter plus the *flgB*p*kan* cassette were PCR amplified using primers P21 & P22 and combined with the vector by Gibson Assembly. The plasmid pSS101 (25 μg DNA linearized with XmaI) was transformed into BhΔ*arcA*. Transformants were selected by plating on 200 μg ml^-1^ kanamycin and confirmed using primers pairs P45 & P46, P47 & P48, and P48 & P49.

The *B*. *hermsii ΔarcC* (*bh0843A*) mutant and complemented strains were constructed with suicide vectors pSS102 and pSS103, respectively (S2 Table). To create a suicide vector to delete *bh0843A* in the BhWT chromosome, approximately 1 kB of DNA 5’ and 0.8 kB 3’ to *bh0843A* was PCR amplified using primers P17 & P25 and cloned into pCR-XL-TOPO (Thermo Fisher Scientific, Inc.). The plasmid was linearized with AccI and amplified by PCR to eliminate *arcC* using primers P26 & P27. The kanamycin resistance gene was amplified from pTA-flgBpKan using primers P28 & P29 and the two PCR products were combined using Gibson Assembly, yielding pSS102. BhWT were transformed with approximately 25 μg of DNA. Transformants were selected in 200 μg ml^-1^ kanamycin and confirmed using primers primer pairs P45 & P46 and P47 & P50. An isogenic clone confirmed to have *arcC* deleted was named BhΔ*arcC*.

Originally, we attempted to complement BhΔ*arcC* by inserting the *arcC* gene and its native promoter downstream of the mutation on the chromosome. To do this, plasmid pSS102 was linearized with BsgI and amplified with primers P23 & P24. The *arcC* gene with 300 bp upstream was PCR amplified with primers P34 & P35 and cloned into pKFSS1 in sites KpnI/PstI yielding pKFSS1::Bh*arcC*. The *B*. *burgdorferi flgB* promoter in front of the *aadA* gene was replaced with the *B*. *hermsii flgB*p sequence by amplifying the BhflgBp with primers P36 & P37 followed by cloning into the plasmid sites FspI/NdeI. The *arcC* and BhflgBp-aadA cassette were amplified from this resulting plasmid with primers P32 & P33 and combined with the PCR-amplified pSS102 by Gibson Assembly, yielding pTOPOcomp1. Transformation of pTOPOcomp1 into Bh*ΔarcC* did not yield any complemented strains. Next, we added more flanking DNA at the 3’ end into the plasmid by PCR-amplifying DNA from *B*. *hermsii* genomic DNA with primers P24 & P38, digested with AflII and BamHI and cloned into the vector with the same enzymes. This resulting plasmid (pTOPOcompEX) did not produce any complemented strains either. Therefore, the suicide vector, pSS103, was created to reintroduce the carbamate kinase gene into the chromosome in its original location with the addition of the streptomycin resistance cassette. To create pSS103, the plasmid TOPOcompEX was linearized with MfeI and PCR-amplified to eliminate the kanamycin-resistance gene with primers P30 & P31 and ligated to itself by Gibson Assembly. BbΔ*arcC* was transformed with pSS103 using 25 μg DNA linearized with AatII and transformants were selected on 50 μg ml^-1^ of streptomycin. Transformants were confirmed by PCR with primer pairs P47 & P51, P46 & P52, and P53 & P54.

To construct the *B*.*burgdorferi* strain containing the *B*. *hermsii arcC* gene, a 1.8 kb fragment was amplified from *B*. *burgdorferi* genomic DNA with primers P35 & P40, digested with XhoI/ClaI and ligated into the vector pPCR-ScriptCm digested with the same restriction enzymes. This vector was then linearized with SpeI and amplified with primers P41 & P42 and gel-purifed. The *B*.*hermsii arcC* and approximately 300 bp upstream was amplified with primers P34 & P35 from genomic DNA. This fragment, as well as PKFSS1, were digested with KpnI/PstI and ligated together to place the *B*. *hermsii arcC* gene and promoter next to the *B*. *burgdorferi flgB*p-*aadA* cassette. The *arcC* and streptomycin-resistance cassettes were amplified from this plasmid with primers P43 & P44 and combined with the linearized pPCR vector containing the *B*. *burgdorferi* flanking DNA by Gibson Assembly. The resulting plasmid, pPCR::Bb-*arcC*in, was linearized with NcoI and 25 ug of DNA was transformed into *B*. *burgdorferi* A3 and selected on plates containing 50μg ml^-1^ streptomycin.

### RNA Extraction and Quantitative Reverse Transcription Polymerase Chain Reaction (qRT-PCR)

RNA samples were prepared for RNA analysis by harvesting cells at 4°C by centrifugation, followed by resuspension of the cell pellet in a Trizol solution and the RNA extracted according to the manufacturer’s instructions (Invitrogen, Grand Island, NY). RNA samples were subsequently treated with TURBO DNase (Ambion, Austin, TX), following the manufacturer’s instructions and using the rigorous protocol. Quantitative RT-PCR was performed using a one-step QuantiTect SYBR Green kit (Qiagen, Hilden, Germany) following the manufacturer’s instructions. To confirm gene deletions and complementation/reconstitution, transcripts for each mutated orf were assayed using the following RNA/primer combinations: *arcA* transcripts were assayed in BbWT, BbΔ*arcA* with P67 & P68 and in BhWT, BhΔ*arcA*, and BhΔ*arcA*-COMP with P57 & P58; *arcC* transcripts were assayed in BhΔ*arcC*, BhΔ*arcC*-RECON and Bb+*arcC* with P63 & P64 (S2 Table). In addition, BbWT and BhWT cultures treated with L-arginine, L-arginine and L-ornithine, and L-ornithine alone were assayed for *arc* operon gene expression using the primers listed in S2 Table in the following combinations: *arcA*_*Bb*_, P67 & P68; *arcB*_*Bb*_ P69 & P70; *arcD*_*Bb*_ P71 & P72; *arcA*_*Bh*_ P57 & P58; *arcB*_*Bh*_ P59 & P60; *arcD*_*Bh*_ P61 & P62; and *arcC*_*Bh*_ P63 & 64. cDNA was synthesized at 42°C for 30 minutes and denatured at 95°C for 15 minutes. Quantitative RT-PCR was performed under standard conditions on a LightCycler (Roche, Indianapolis, IN). The LightCycler software was used for the analysis of fold changes and *rpoD* normalization (P73 & P74 for *B*. *burgdorferi*; P65 & 66 for *B*. *hermsii*).

### Protein expression and purification, SDS-PAGE Gel Electrophoresis and Immunoblot analysis

Proteins encoding the borrelial arginine utilization genes, arginine deiminase (*arcA*, *bb0841/bh0841*), ornithine transcarbamylase (*arcB*, *bb0842/bh0842*), and carbamate kinase (*arcC*, *bh0843A*) were expressed in *Escherichia coli* BL-21 DE3 (Invitrogen). Each ORF was codon optimized for *E*. *coli*, synthesized and subcloned into pET45b or pET30a (Genscript) (S1 Table). Chemically competent *E*. *coli* were transformed with 5 μg of plasmid DNA and transformants were selected on LB agar with100 μg ml^-1^ carbenicillin (Sigma). Colonies were inoculated into 10 ml LB broth and incubated shaking overnight at 34°C, then transferred to 500 ml overnight express and incubated shaking for 48 hours at 34°C. Cultures were harvested by HIS tag purification according to a previously published protocol [93]. Following protein purification, polyclonal antibodies against each protein were commercially generated (Genscript).

*B*. *burgdorferi* and *B*. *hermsii* cultures were treated with L-arginine, L-arginine and L-ornithine, and L-ornithine alone as described above. Samples were prepared for immunoblot analysis as previously described [93]. Briefly, cells were harvested by centrifugation (8,000 x g, 10 min, 4^○^C) and washed with HN buffer followed by lysis at 99°C. Protein concentration in each whole cell lysate was quantified by absorbance and 40 μg of total protein or 2 μg purified protein was loaded into each lane of an Any kD polyacrylamide gel (Biorad, Hercules, CA). Proteins were transferred to nitrocellulose membranes (Biorad) and immunoblot analysis was performed as previously described [89]. Primary antibodies were used at the following concentrations: α-ArcA_Bb_ (1:500), α-ArcA_Bh_ (1:500), α-ArcB_Bh_ (1:500), α-ArcC_Bh_ (1:500), α-ArcD (1:1000) and α-eno_Bb_ polyclonal antiserum (1,1000) (Genscript) [94]. A horseradish peroxidase conjugated recombinant protein A (Thermo Fisher Scientific, Inc.) was used as a secondary antibody at 1:1000 and proteins were visualized with the ECL Plus Western Blotting Detection Reagents (GE Healthcare, Pittsburgh, PA).

### Amino acid analysis

Mouse plasma and culture supernatants were analyzed for L-arginine, L-citrulline and L-ornithine concentrations by high pressure liquid chromatography as previously described [94]. Mouse plasma and culture supernatants were deproteinized by filtration through a 10 kDa centrifugal filter unit (Millipore, Burlington, MA) followed by extraction of the flow-through with 0.6 N trichloroacetic acid. Following TCA extraction, 50 μl of supernatant was derivatized in 100 μl of a 6/1/1/1 (v/v/v/v) solution of methanol/ethanol/triethylamine/ultra-pure water/PITC for 20 minutes. Samples were evaporated and resuspended in 150 μl mobile phase A and filtered through a 0.45 μm nylon membrane syringe filter (GE Healthcare). Mobile phase A consisted of 0.12 M sodium acetate and 2.5 μM EDTA buffer with 2.5% acetonitrile (pH 6.5) and mobile phase B consisted of 15% methanol, 45% acetonitrile, and 40% ultra-pure water. Prior to use, mobile phases were filtered using a 0.2 μm filter with a vacuum followed by ultrasonic degassing. Separations were conducted using a Zorbax Eclipse Plus C18 HPLC column, 4.6 x 150 mm i.d., 5μm particle size (Agilent). Gradient elution was set to 0 to 10 min, 100% A to 75% A; 10 to 11 min, 75% A to 30% A; 11 to 15 min, 30% A to 25% A; 15 to 17 min, 25% A to 10% A; 17 min to 20 min, 10% A to 100% A and 20 μl sample injections were used. L-arginine monohydrochloride, L-citrulline and L-ornithine monohydrochloride (Sigma) were used as standards for peak identification and quantification.

### Analysis of infectivity of *arc* mutants in a murine model

To test the infectivity of the *B*. *burgdorferi* Δ*arcA* (BbΔ*arcA*) and the *B*. *burgdorferi* carbamate kinase knock-in (Bb+*arcC*_*Bh*_*)* mutants, compared to wild-type, each strain was grown to mid-logarithmic phase and three groups of five female IRW mice were obtained from the Rocky Mountain Laboratories Veterinary Branch (RMVB) breeding facility. Research using IRW mice was first published by Chesebro et al. in 1983 [95]. IRW mice are an inbred colony that is maintained at RML and have been used in numerous published studies [95–101]. Each mouse was inoculated by intraperitoneal injection with an inoculum of 5 x 10^4^ cells grown in BSKII. Inocula were plated to confirm dose and plasmid content. Three weeks post-inoculation, blood was collected by submandibular bleeds to perform serology on each animal and confirm infection. Seven to 14 days later, mice were anesthetized by inhalation of isoflurane and for experiments where amino acids were analyzed, approximately 500–700 μl blood was drawn by cardiac puncture immediately prior to cervical dislocation. Tissues from the ear, bladder, and tibiotarsus joint tissues were then immediately removed and cultured in BSK II containing a *Borrelia*-specific triple antibiotic mixture (HiMedia Laboratories, West Chester, PA) to test for spirochete positive tissue.

To test whether BbΔ*arcA* or Bb+*arcC*_*Bh*_ could be acquired by *Ixodes scapularis*, approximately 50 larval ticks were fed to repletion on individual mice. Two naïve mice, for each bacterial strain, were infected by intraperitoneal needle inoculation and were confirmed to be infected by ear punch biopsy, 3 weeks post inoculation. Larval *I*. *scapularis* were obtained from Oklahoma State University. All tick culturing experiments utilized BSK II containing the *Borrelia*-specific triple antibiotic mix (HiMedia Laboratories) unless otherwise stated. Following feeding, ticks were decontaminated prior to plating: ticks were washed for 3 min in 3% H_2_O_2_, 3 min in 70% ethanol, and then finally rinsed in sterile water. Decontaminated ticks were individually homogenized with a mortar and pestle in 0.5 ml BSK II. Serial dilutions were performed on homogenates and dilutions were plated on BSK II agar. Homogenization and plating of ticks occurred within one day after ticks had fed to repletion.

Initially, BhWT and four BhΔ*arc* mutants were assessed for infectivity in the murine model using 5 groups of 5 IRW mice, each inoculated with 500–1000 spirochetes by intraperitoneal injection. The experiment was performed in duplicate for a total of 10 mice per strain. Each mouse was assessed for spirochetemia daily by extracting approximately 5 μl of blood by a submandibular bleed followed by microscopic examination of blood smears. Upon confirmation of a spirochetemic infection (as evidenced by positive blood smears visualized by dark field microscopy), each mouse was anesthetized by inhalation of isoflurane and, while under anesthesia, approximately 500–700 μl of blood was removed prior to humane euthanasia by cervical dislocation. 10–100 μl of whole blood was diluted in BSK II and plated as previously described [91].

To quantify differences in the relapse profile of BhWT, BhΔ*arcA*, and BhΔ*arcA*-COMP four IRW mice were inoculated with 1000 spirochetes by intraperitoneal injection and spirochetes levels were quantified by QPCR daily from submandibular bleeds taken on days 2–13 as described by McCoy et al. [62]. Briefly, a 5 μl drop of blood was placed into 95 μl of SideStep Lysis and Stabilization Buffer (Agilent Technologies) in duplicate and stored at -80°C. Upon completion of the experiment, the samples, including uninfected blood samples, were thawed and diluted 1∶10 in sterile water and 3 μl were used as template in triplicate in QPCR using the Stratagene Brilliant II QPCR Master Mix (Agilent Technologies) with a probe and primer set to the *B*. *hermsii flaB* gene. To quantify the total number of spirochetes per ml of blood per mouse, a standard curve using a 10-fold serial dilution of a known number of spirochetes spiked into the Stabilization buffer along with 5 μl of uninfected blood was processed similarly.

To test whether the BhΔ*arc* mutants can be acquired by *Ornithodoros hermsi*, approximately 40–50 second stage nymphs were fed to repletion on individual mice once they were spirochetemic. The maintenance of the *O*. *hermsi* tick colony has been previously described [62]. Following feeding, ticks were decontaminated prior to plating: ticks were washed for 3 min in 3% H_2_O_2_, 3 min in 70% ethanol, and then finally rinsed in sterile water. Individual ticks were then homogenized with a mortar and pestle in 0.5 ml BSK II with 12% rabbit serum plus the *Borrelia*-specific triple antibiotic mix (HiMedia Laboratories). Serial dilutions were performed on homogenates and dilutions were plated as previously described [91]. Homogenization and plating of ticks occurred within one day after ticks had fed to repletion. The number of spirochetes in the blood at the time of tick feeding was enumerated by plating as previously described [91].

### Statistical analysis

To test for significant differences in spirochetal growth due to the addition of L-arginine and ornithine, as well as to test for differences in ADI mutant growth, a repeated measures ANOVA with a Dunnett correction for multiple comparisons was employed. To test for significant differences in ADI transcript levels as well as differences in L-arginine, L-citrulline, and L-ornithine utilization, two-way ANOVA’s with Tukey’s multiple comparison test were conducted for each respective experiment. To test for significant differences in L-arginine, citrulline or ornithine utilization in mouse blood during infection, individual one-way ANOVA’s with Tukey’s multiple comparison test were conducted.

## Supporting information

S1 Fig Unrooted phylogenetic tree of arginine deiminase (*arcA*) sequences. The tree was based on inferred amino acid sequences (lengths ranged from 407 to 471 amino acids) and constructed using maximum likelihood analysis, RAxML with bootstrap analysis (1000 replicates). The values at nodes represent RAxML bootstrap values.
(TIF)

S2 Fig Unrooted phylogenetic tree of ornithine transcarbamylase (*arcB*) sequences. The tree was based on inferred amino acid sequences (length ranged from 306–350) and constructed using maximum likelihood analysis, RAxML with bootstrap analysis (1000 replicates). The values at nodes represent RAxML bootstrap values.
(TIF)

S3 Fig Unrooted phylogenetic tree of the putative L-arginine/L-ornithine antiporter (*arcD*) sequences. The tree was based on inferred ArcD amino acid sequences (lengths ranged from 462 to 483 amino acids) and constructed using maximum likelihood analysis, RAxML with bootstrap analysis (1000 replicates). The values at nodes represent RAxML bootstrap values.
(TIF)

S4 Fig Unrooted phylogenetic tree of carbamate kinase (*arcC*) sequences. The tree was based on inferred amino acid sequences (lengths ranged from 310 to 332) and constructed using maximum likelihood analysis, RAxML with bootstrap analysis (1000 replicates). The values at nodes represent RAxML bootstrap values.
(TIF)

S5 Fig Genetic organization of the ADI pathway in wild-type and mutant strains used in this study. A) *B*. *burgdorferi* and B) *B*. *hermsii*.
(TIF)

S6 Fig Polyclonal antibodies generated against *B*. *hermsii* arginine deiminase and ornithine transcarbamylase react with recombinant ArcA and ArcB, respectively, from both *B*. *burgdorferi* and *B*. *hermsii*. A) Top panel: Densitometry analysis of recombinant ArcA (as probed by western blot, pictured in bottom panel). Asterisks indicate statistical significance < 0.05 in a one-way ANOVA. Bottom panel: Lanes 1–5 contain 80, 40, 20, 10 and 5 μg, respectively, purified, recombinant ArcA_Bb_. Lanes 7–11 contain 80, 40, 20, 10 and 5 μg respectively, purified, recombinant ArcA_Bh_ The primary antibody was a polyclonal anti-ArcA (1:500, generated against ArcA_Bh_), secondary was HRP-rec-Protein A (1:1000). B) Top panel: Densitometry analysis of recombinant ArcB (as probed by western blot, pictured in bottom panel). Asterisks indicate statistical significance < 0.05 in a one-way ANOVA. Bottom panel: Lanes 1–5 contain 80, 40, 20, 10 and 5 μg, respectively, purified recombinant ArcB_Bb_. Lanes 7–11 contain 80, 40, 20, 10 and 5 μg respectively, purified recombinant ArcB_Bh._ The primary antibody was a polyclonal anti-ArcB (1:500, generated against ArcB_Bh_), secondary was HRP-rec-Protein A (1:1000). Western blot signal intensities were determined using FIJI software.
(TIF)

S7 Fig Alteration of the ADI by targeted mutagenesis did not affect growth of *B*. *burgdorferi* or *B*. *hermsii* or alter expression levels of Arc proteins in *B*. *burgdorferi*. In vitro growth of ADI mutants in A) *B*. *burgdorferi* and B) *B*. *hermsii*. C) Expression of Arc proteins in the presence and absence of *arcC*_Bh_ on the *B*. *burgdorferi* chromosome.
(TIF)

S1 Table Bacterial strains and plasmids used in this study. (DOCX)

S2 Table Primers used in this study. (DOCX)
