# Correction: The arginine deaminase system plays distinct roles in *Borrelia burgdorferi* and *Borrelia hermsii*

**DOI:** 10.1371/journal.ppat.1010549

**Published:** 2022-05-10

**Authors:** Crystal L. Richards, Sandra J. Raffel, Sébastien Bontemps-Gallo, Daniel P. Dulebohn, Tessa C. Herbert, Frank C. Gherardini

The word "deiminase" is misspelled in the article title. The correct title is: The arginine deiminase system plays distinct roles in *Borrelia burgdorferi* and *Borrelia hermsii*.

The correct citation is: Richards CL, Raffel SJ, Bontemps-Gallo S, Dulebohn DP, Herbert TC, Gherardini FC (2022) The arginine deiminase system plays distinct roles in *Borrelia burgdorferi* and *Borrelia hermsii*. PLoS Pathog 18(3): e1010370. https://doi.org/10.1371/journal.ppat.1010370
