# Supplementary figures and images for: The arginine deaminase system plays distinct roles in Borrelia burgdorferi and Borrelia hermsii
Source: PLoS Pathog. 2022 Mar 14;18(3):e1010370. doi: 10.1371/journal.ppat.1010370 (PMC8947608; doi:10.1371/journal.ppat.1010370)

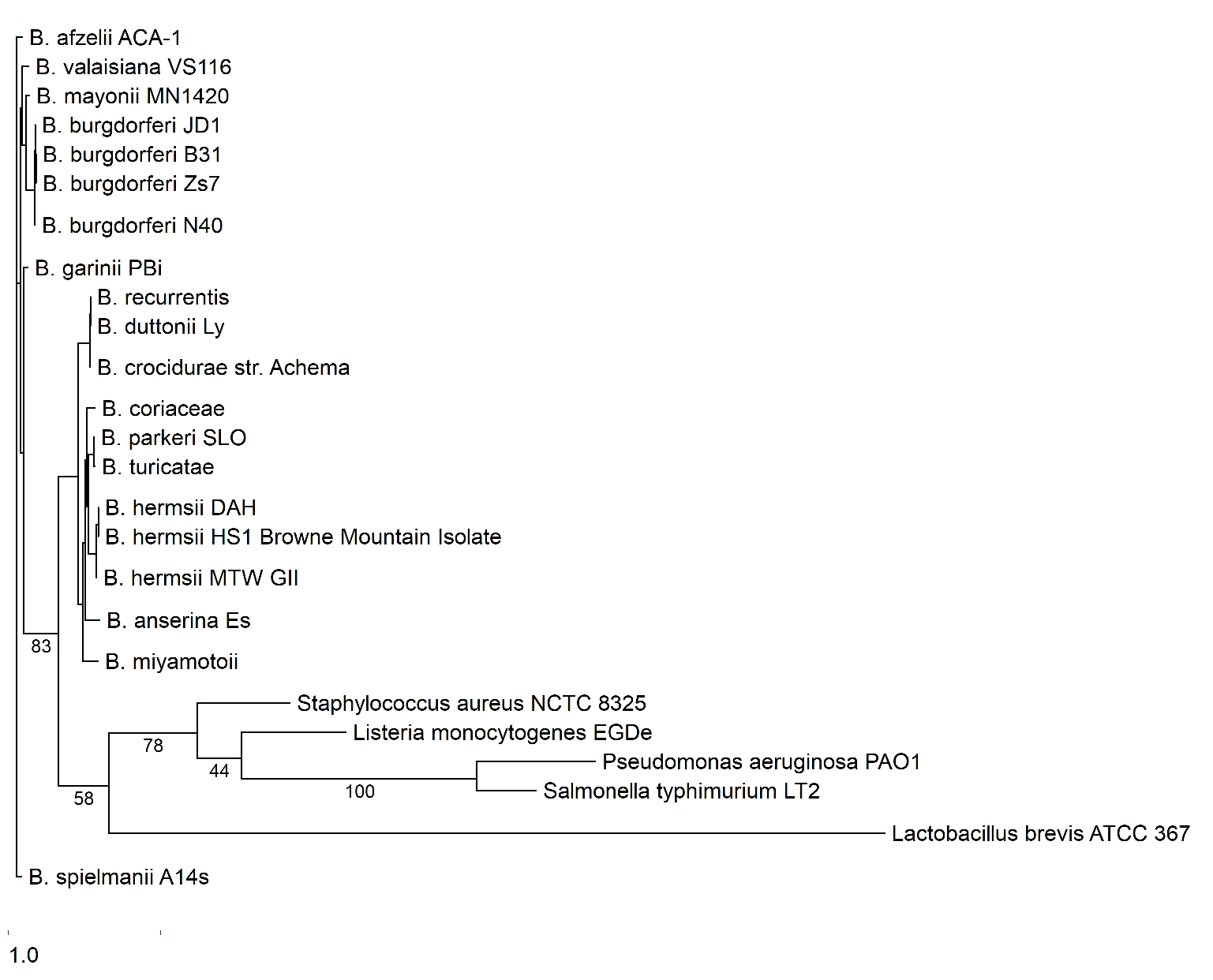

Supplement: S1 Fig — The tree was based on inferred amino acid sequences (lengths ranged from 407 to 471 amino acids) and constructed using maximum likelihood analysis, RAxML with bootstrap analysis (1000 replicates). The values at nodes represent RAxML bootstrap values. (TIF) [file ppat.1010370.s001.tif]

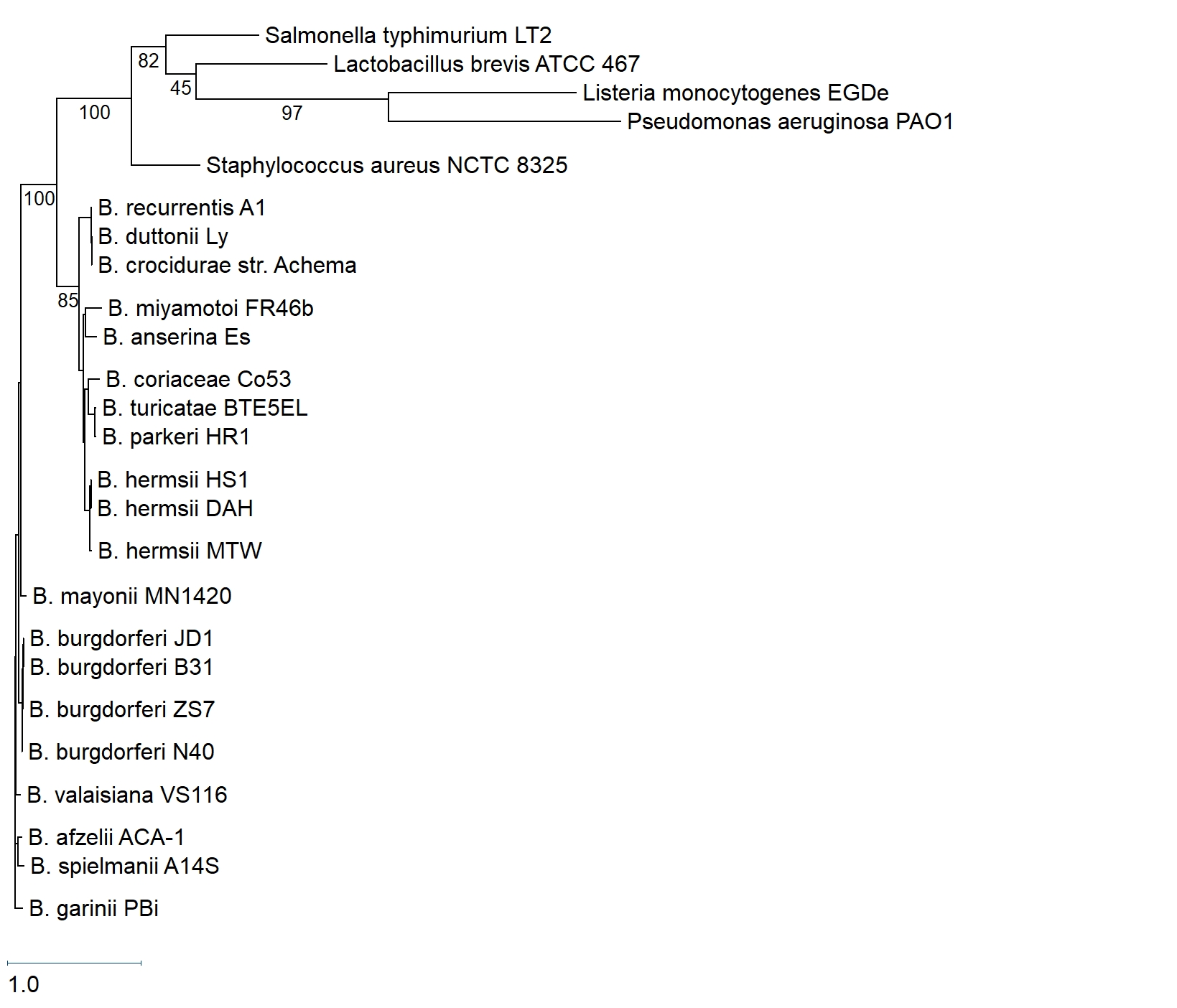

Supplement: S2 Fig — The tree was based on inferred amino acid sequences (length ranged from 306–350) and constructed using maximum likelihood analysis, RAxML with bootstrap analysis (1000 replicates). The values at nodes represent RAxML bootstrap values. (TIF) [file ppat.1010370.s002.tif]

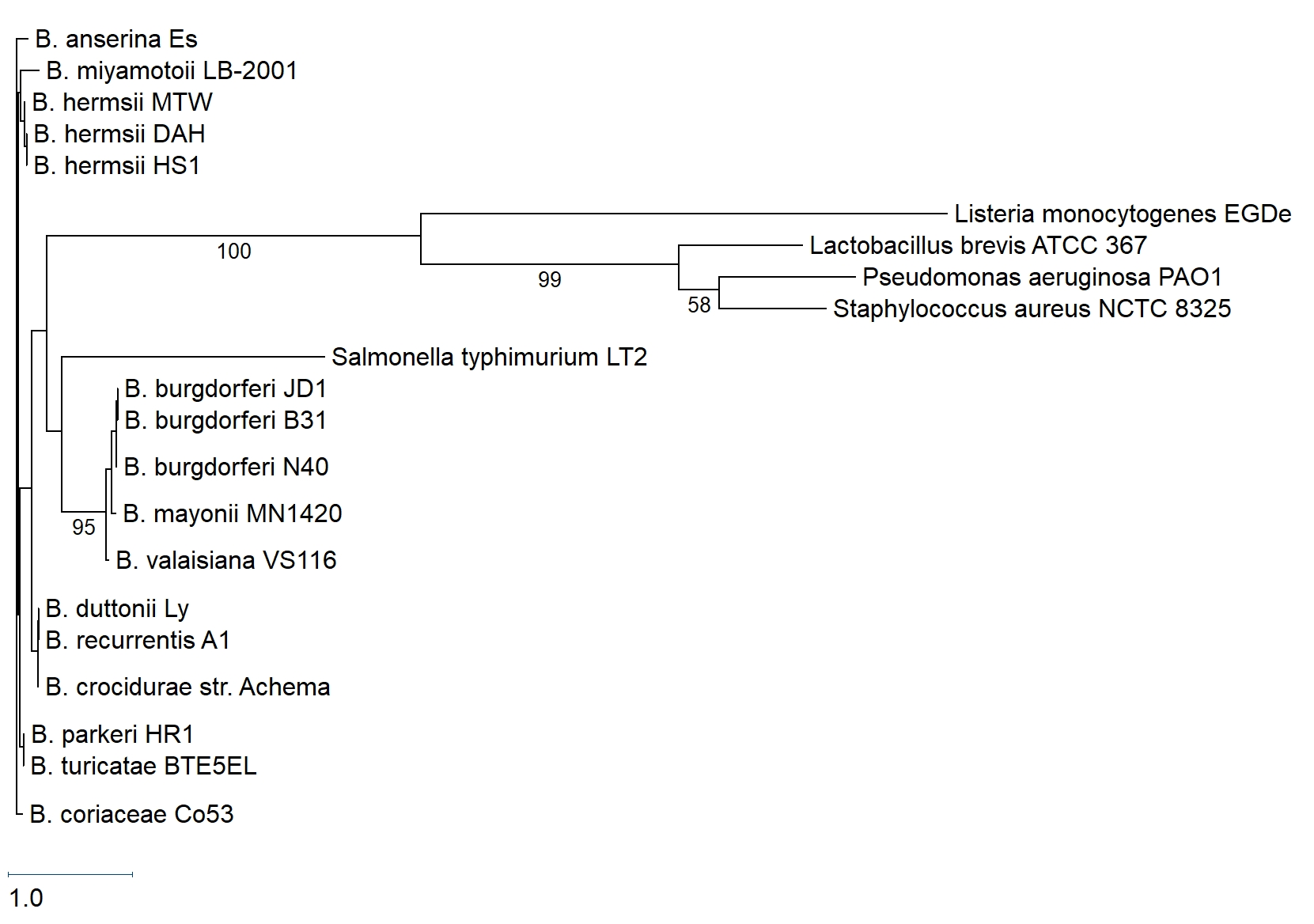

Supplement: S3 Fig — The tree was based on inferred ArcD amino acid sequences (lengths ranged from 462 to 483 amino acids) and constructed using maximum likelihood analysis, RAxML with bootstrap analysis (1000 replicates). The values at nodes represent RAxML bootstrap values. (TIF) [file ppat.1010370.s003.tif]

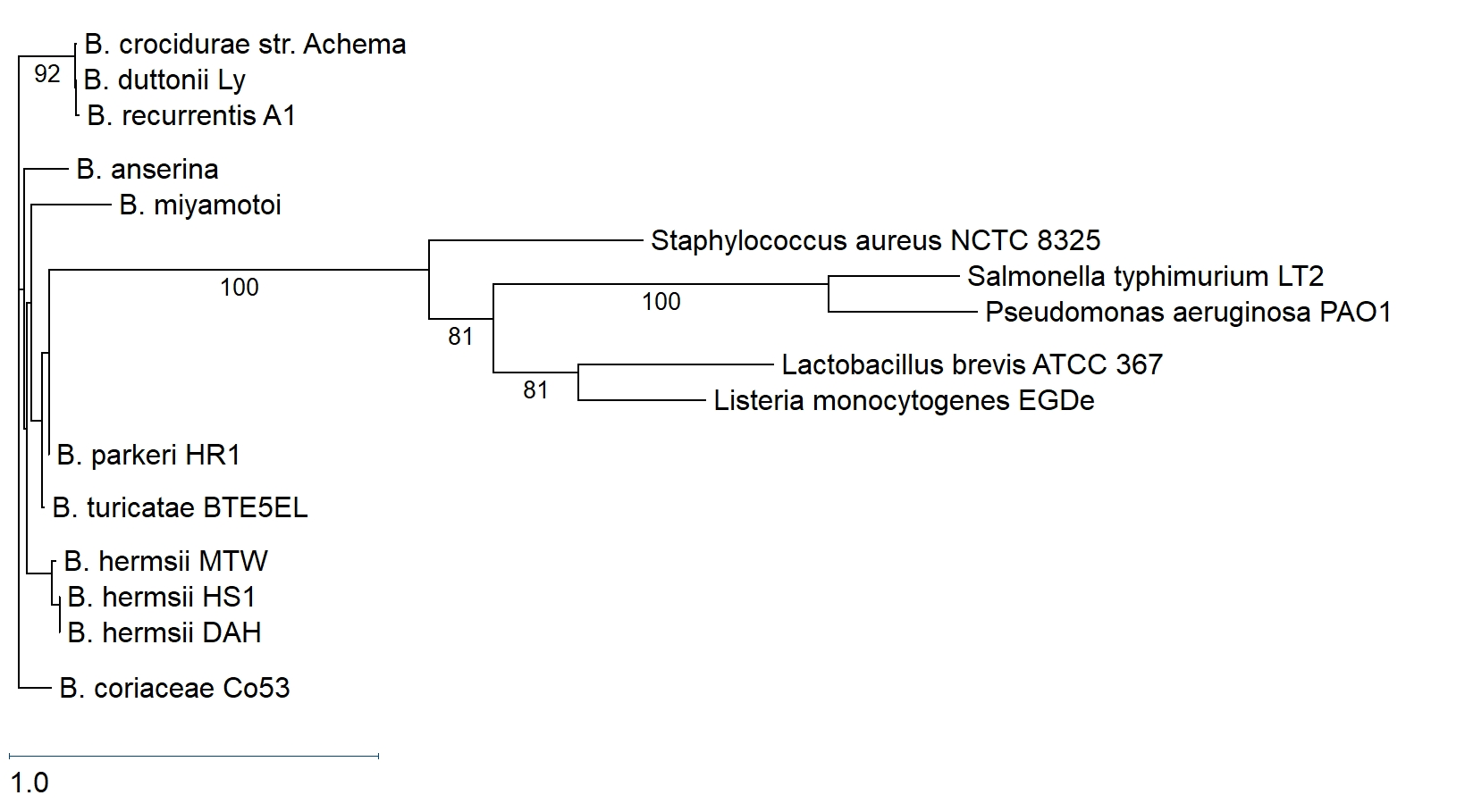

Supplement: S4 Fig — The tree was based on inferred amino acid sequences (lengths ranged from 310 to 332) and constructed using maximum likelihood analysis, RAxML with bootstrap analysis (1000 replicates). The values at nodes represent RAxML bootstrap values. (TIF) [file ppat.1010370.s004.tif]

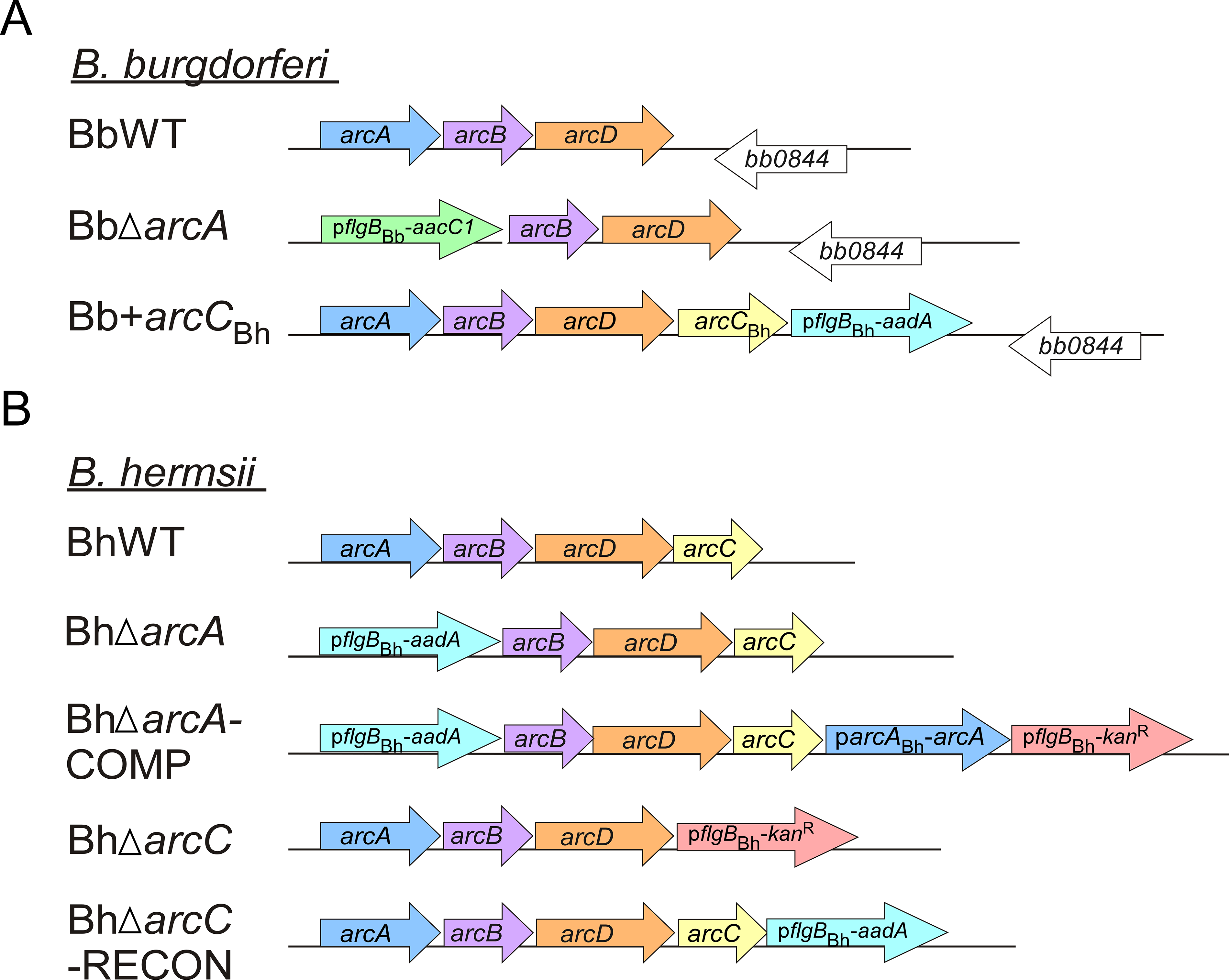

Supplement: S5 Fig — A) B. burgdorferi and B) B. hermsii. (TIF) [file ppat.1010370.s005.tif]

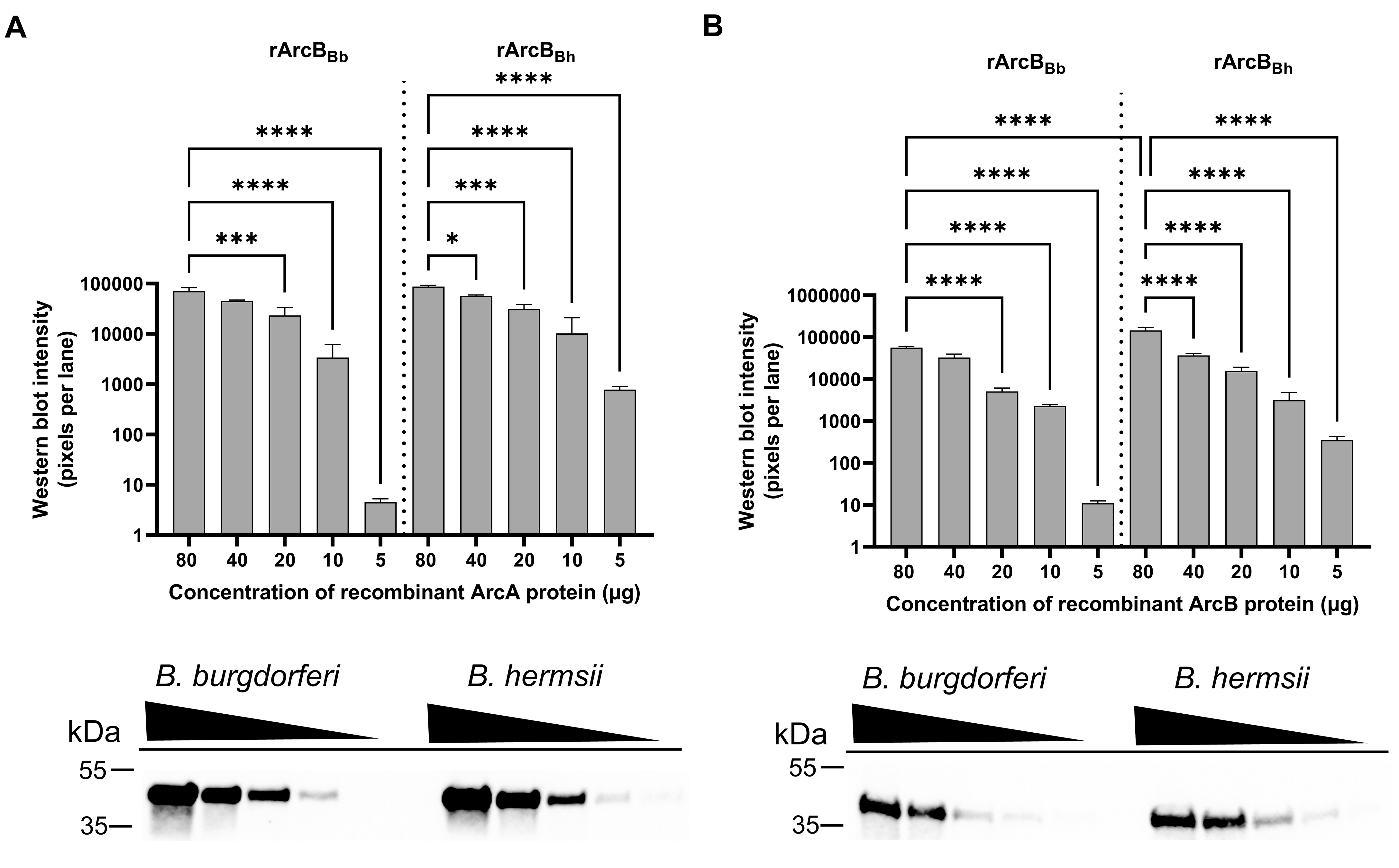

Supplement: S6 Fig — A) Top panel: Densitometry analysis of recombinant ArcA (as probed by western blot, pictured in bottom panel). Asterisks indicate statistical significance < 0.05 in a one-way ANOVA. Bottom panel: Lanes 1–5 contain 80, 40, 20, 10 and 5 μg, respectively, purified, recombinant ArcABb. Lanes 7–11 contain 80, 40, 20, 10 and 5 μg respectively, purified, recombinant ArcABh The primary antibody was a polyclonal anti-ArcA (1:500, generated against ArcABh), secondary was HRP-rec-Protein A (1:1000). B) Top panel: Densitometry analysis of recombinant ArcB (as probed by western blot, pictured in bottom panel). Asterisks indicate statistical significance < 0.05 in a one-way ANOVA. Bottom panel: Lanes 1–5 contain 80, 40, 20, 10 and 5 μg, respectively, purified recombinant ArcBBb. Lanes 7–11 contain 80, 40, 20, 10 and 5 μg respectively, purified recombinant ArcBBh. The primary antibody was a polyclonal anti-ArcB (1:500, generated against ArcBBh), secondary was HRP-rec-Protein A (1:1000). Western blot signal intensities were determined using FIJI software. (TIF) [file ppat.1010370.s006.tif]

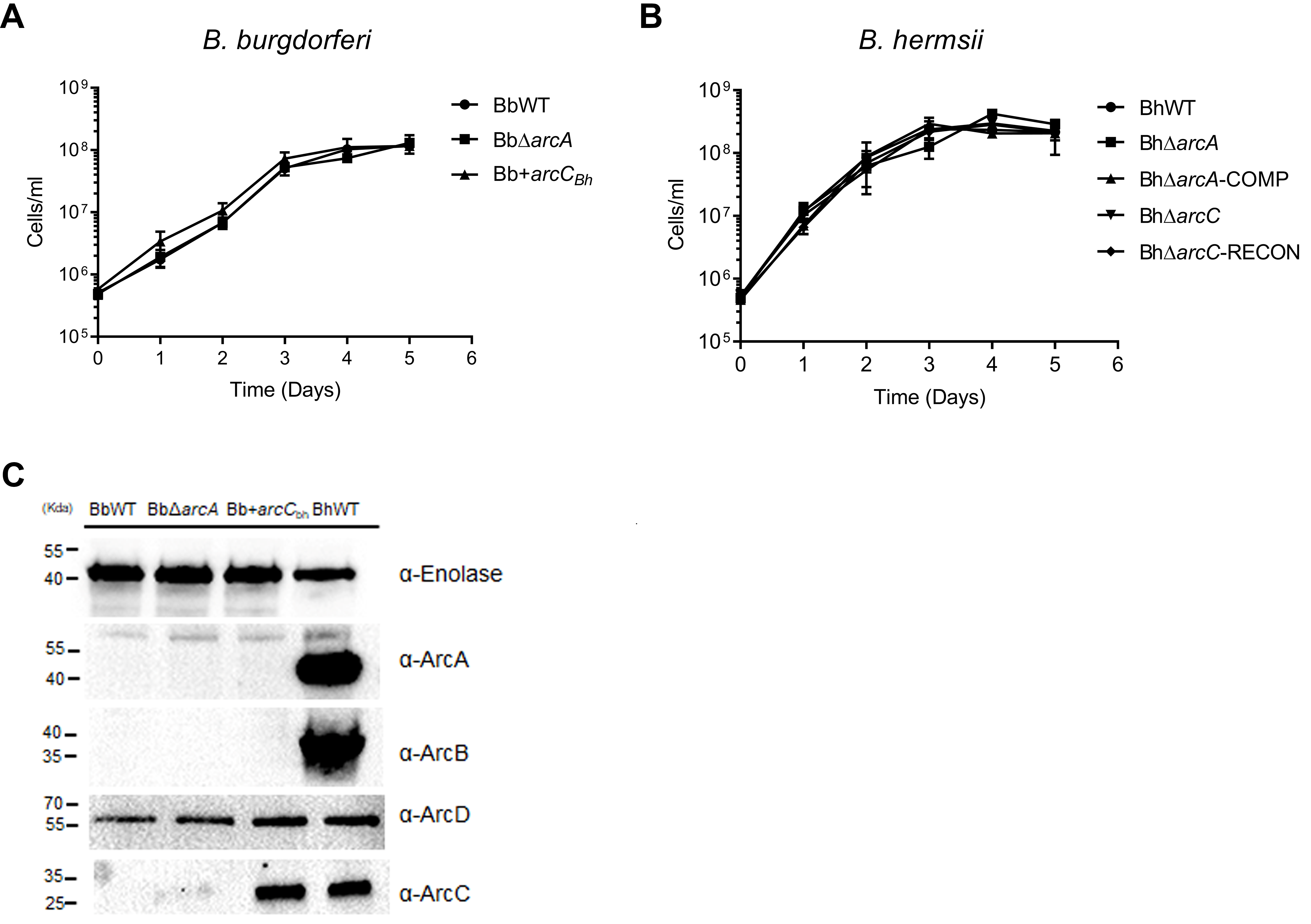

Supplement: S7 Fig — In vitro growth of ADI mutants in A) B. burgdorferi and B) B. hermsii. C) Expression of Arc proteins in the presence and absence of arcCBh on the B. burgdorferi chromosome. (TIF) [file ppat.1010370.s007.tif]
