# Supplementary material for: The arginine deaminase system plays distinct roles in Borrelia burgdorferi and Borrelia hermsii
Source: PLoS Pathog. 2022 Mar 14;18(3):e1010370. doi: 10.1371/journal.ppat.1010370 (PMC8947608; doi:10.1371/journal.ppat.1010370)
Supplement: S1 Table — (DOCX) [file ppat.1010370.s008.docx]

**S1 Table. Bacterial strains and plasmids used in this study**

| **Strains** | **Reference** |
| --- | --- |
| *B. burgdorferi* B31-A3 (BbWT) | [1] |
| BbΔ*arcA* | This study |
| Bb+*arcC*_Bh_ | This study |
| *B. hermsii* DAH (BhWT) | [2] |
| BhΔ*arcA* | This study |
| BhΔ*arcA*-COMP | This study |
| BhΔ*arcC* | This study |
| BhΔ*arcC*-RECON | This study |
| *Escherichia coli* TOP 10 | Invitrogen |
| **Plasmids** |  |
| pPCRscript CAM SK+ | Invitrogen |
| pCR102 (pPCRscript CAM SK+::Δ*arcA*_Bb_::*aacC1*) | This study |
| pBSV2G | [3] |
| pKFSS1 | [4] |
| pTOPOXL | Invitrogen |
| pSS100 (pTOPOXL::Δ*arcA*_Bh_::*aadA*) | This study |
| pZero Blunt TOPO | Invitrogen |
| pSS101 (pzero-blunt TOPO::*arcA*_Bh_::kan^R^) | This study |
| pTA-flgBpKan | This study |
| pSS102 (pTOPOXL::Δ*arcC*_Bh_::kan^R^) | This study |
| pSS103 (pTOPOXL::*arcC*_Bh_::*aadA*) | This study |
| pTOPOcompEX | This study |
| pSS104 (pPCR::*arcC*_Bh_*::aadA*) | This study |
| pET-45b::*arcA*_Bb_ | Genscript |
| pET-45b::*arcB*_Bb_ | Genscript |
| pET-30a+::*arcA*_Bh_ | Genscript |
| pET-30a+::*arcB*_Bh_ | Genscript |
| pET-45b::*arcC*_Bh_ | Genscript |

**References**

1. Elias AF, Stewart PE, Grimm D, Caimano MJ, Eggers CH, Tilly K, et al. Clonal polymorphism of *Borrelia burgdorferi* strain B31 MI: implications for mutagenesis in an infectious strain background. Infect Immun. 2002;70:2139-50.

2. Hinnebusch BJ, Barbour AG, Restrepo BI, Schwan TG. Population structure of the relapsing fever spirochete *Borrelia hermsii* as indicated by polymorphism of two multigene families that encode immunogenic outer surface lipoproteins. Infect Immun. 1998;66(2):432-40.

3. Elias AF, Bono JL, Kupko JJ, 3rd, Stewart PE, Krum JG, Rosa PA. New antibiotic resistance cassettes suitable for genetic studies in *Borrelia burgdorferi*. J Mol Microbiol Biotechnol. 2003;6(1):29-40.

4. Frank KL, Bundle SF, Kresge ME, Eggers CH, Samuels DS. *aadA* confers streptomycin resistance in *Borrelia burgdorferi*. J Bacteriol. 2003;185:6723-7.
