# Supplementary material for: The arginine deaminase system plays distinct roles in Borrelia burgdorferi and Borrelia hermsii
Source: PLoS Pathog. 2022 Mar 14;18(3):e1010370. doi: 10.1371/journal.ppat.1010370 (PMC8947608; doi:10.1371/journal.ppat.1010370)
Supplement: S2 Table — (DOCX) [file ppat.1010370.s009.docx]

**S2 Table. Primers used in this study**

| Primer | Primer sequence 5' to 3' |
| --- | --- |
| P1 | ACTGCTGGTACCTGATTAAAATTTGTTTTTTTTTC |
| P2 | ACTGCTCTCGAGCGATTTCCCCCTTTATGAAAATTG |
| P3 | ACTGCTAAGCTTTAATATTTAAGCATGTTTAAGC |
| P4 | ACTGCTGAATTCATAGCACAGCCTTTCTTCAATAAAG |
| P5 | ACTGCTCTCGAGTAATACCCGAGCTTCAAGGA |
| P6 | ACTGCTAAGCTTTTAGGTGGCGGTACTTGGGTCGAT |
| P7 | GGCACAAGAGAATAATAAAGTGTGTG |
| P8 | GTTTTAGGGCGACTGCC |
| P9 | ATCAACCACCAACCTTTACAATCAAG |
| P10 | GGCATTGCCACCGCGCTCATCAATCTCCTC |
| P11 | TCCTGTTTCTGGATCTATGATATTGGG |
| P12 | CCCAAAGTCCCAACCATTACAGC |
| P13 | CACCGCTTCCCTCATAACAAACACCCCC |
| P14 | AAGGTAGTCGGCAAATAATTTAACGGAGGATATATTTATG |
| P15 | ATGAGGGAAGCGGTGATCGC |
| P16 | TTATTTGCCGACTACCTTGGTGATCTC |
| P17 | ATTGGATTGGGTGCTGGTGTGGGAAC |
| P18 | TGGCTCTTGCAATCTCATACTTTCCG |
| P19 | CGGATATCTAAATATCTTCCCTTATTAAAGGCATGGAC |
| P20 | GATCTCGAGTATGTGCAAGATGTTGCATGTC |
| P21 | ATGGGGTTTCAAATGAAGTATGTGCAAGATGTTGCATGTC |
| P22 | CATTATGCCACTAAACGTGTTACAACCAATTAACCAATTCTG |
| P23 | CTTCATTTGAAACCCCATTTAACACTTAACAAAACATGATAGC |
| P24 | GTTTAGTGGCATAATGGATGTTGATGCACAAACTGGGGG |
| P25 | ATTGGATTGGGTGCTGGTGTGGGAAC |
| P26 | GCACTGGGACTGGTCTAAATGATTGGCG |
| P27 | TATGGCTCATACATATCTCCTAAATTATAAATACAC |
| P28 | GTTTTTCTAAGGAATTATAATAGCTAGATCAATTGAGAAG |
| P29 | GGAGATATGTATGAGCCATATTCAACGGG |
| P30 | GCTATTATAATTCCTTAGAAAAACTCATCGAGCATC |
| P31 | GTGTATTTATAATTTAGGAGATATGTATGAGTAAAAGAATTGTAGTCAG |
| P32 | ACATATCTCCTAAATTATAAATACACATTTGCCATTATCAC |
| P33 | ATGGGGTTTCAAATGAAGGATGTGTATAGGGGTTGTTGTGC |
| P34 | CATTATGCCACTAAACTTATTTGCCGACTACCTTGGTG |
| P35 | GCTGGTACCGATGTGTATAGGGGTTGTTGTGC |
| P36 | TTATCTGCAGCTATCCAGTTATCATTGTTCCTTCC |
| P37 | GGGATATCGTTAAAGAAAATTGAAATAAACTTG |
| P38 | ATTCATATGAACACCCTCTATATCAC |
| P39 | CGGATCCTGGCTCTTGCAATCTCATACTTTCCG |
| P40 | AGGGGGTGTTTGTTATGCAG |
| P41 | TCTGCAAAAGCATCGTGTTC |
| P42 | TGTGCTATTATGGGCATGGA |
| P43 | TCACCCATGGATACCCAAAC |
| P44 | GTGTATGGGCAAAGGGAAGA |
| P45 | CACCAACCAAGCTCCAAAAT |
| P46 | ACTGCTGTTGATCGGGTAGC |
| P47 | TCCCTACCTTCTCCGGACTT |
| P48 | CAAATGCTGTTCATGCTGCT |
| P49 | TCATGCTAAATGGCTGACCA |
| P50 | TGGTGCAAGAGAACAATGGA |
| P51 | CCCTTGAAAGCTCGCTAGAC |
| P52 | TGCAGGAATTGAAGTGCAAA |
| P53 | TTGAGCCCATTTGATTACCC |
| P54 | GGGGGTGCTTATGGGATTAT |
| P55 | CCGGTTACAGTTCCACCAAT |
| P56 | GGAAAGATTGGCCCTAAAAGA |
| P57 | TCCAAGCACCCTCAAATCTC |
| P58 | CTTCTCGAGCTGGAGTGGGAACTATGGCTTC |
| P59 | TAGTAATCGATCAATACAGGCAAAAAATGAGGCAGAGG |
| P60 | CATATCTCCTAAACTAAAAACTTAAATAAATGTTAGC |
| P61 | CGGCAAATAATAGAAATATTCTTAAAAAAAGTAGAAGG |
| P62 | AAGTTTTTAGTTTAGGAGATATGTATGAG |
| P63 | AAGAATATTTCTATTATTTGCCGACTACC |
| P64 | CGGATTCAGTCGTCACTCATGGTG |
| P65 | AGTTTATGGATGCGGGGCTC |
| P66 | ACTACAACAGGTATGTATGAAGAGACAC |
| P67 | CCATCATTCCACTGTTCTCTCGC |
| P68 | TGAGGTGGCAATACAAGAACACG |
| P69 | GGAAGAGGCATAAATTCCGTCAGCC |
| P70 | ATCACTGTGTGGCTTCAGGC |
| P71 | TCAGCCCGTCATACTTGAAGC |
| P72 | TGGTATGAGTCAAGGGATGATAGGG |
| P73 | ATACCTCCTCCTCCACACGC |
| P74 | ACTAATGAACAGTAAGTTTTATGGTGAATCTC |
| P75 | CTTCTTAACCATCATATCTCTCCTAGTCC |
